# Optimal Integrated Plant for Biodegradable Polymer Production

**DOI:** 10.1021/acssuschemeng.2c05356

**Published:** 2023-02-02

**Authors:** José E. Roldán-San Antonio, Mariano Martín

**Affiliations:** Department of Chemical Engineering, University of Salamanca, Plaza Caídos 1-5, Salamanca37008, Spain

**Keywords:** sawdust, sludge, manure, biogas, biodegradable polymer, biodiesel, circular economy, mathematical optimization

## Abstract

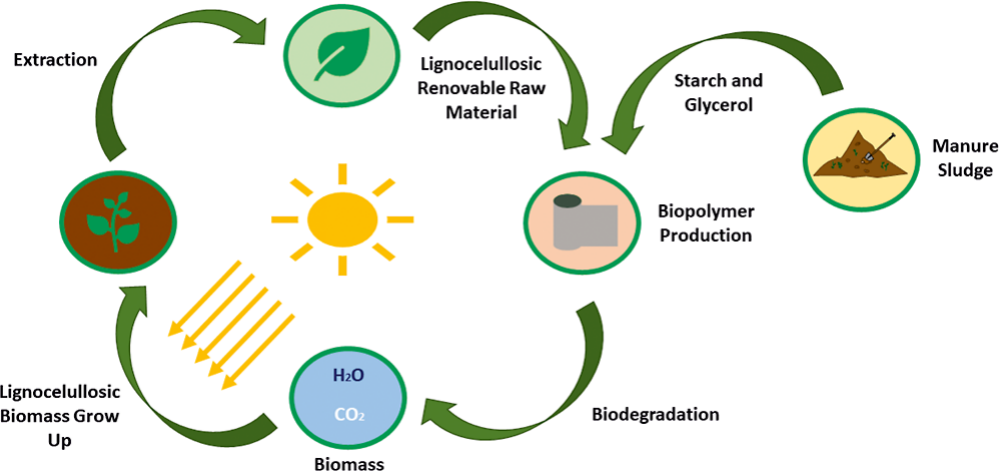

An integrated facility for the production of biodegradable polymers from biomass residues has been developed. Lignocellulosic residues (sawdust), CO_2_, and organic waste such as manure or sludge are the raw materials. Manure and sludge are digested to provide the nutrients needed to grow algae. Algae are used in full to oil and starch production. The oil is transesterified with methanol generated via biogas dry reforming to obtain biodiesel and glycerol. The starch is used together with glycerol and the pretreated sawdust for the production of the biodegradable polymer. A mathematical optimization approach is used to identify the best use of each resource and the optimal operation of the integrated facility for each case. 4732 kt/yr of manure or 4653 kt/yr of sludge was processed to produce 354 kt/yr of biopolymer and 84 Mgal/yr of fatty acid methyl ester, capturing 2.47 kg of CO_2_ per kg of biopolymer with production costs of 0.89 and 0.95 $/kg, respectively, and an investment capital of 717 and 712 M$, respectively.

## Abstract 2

Optimization of integrated plant for biopolymer and biodiesel production with self-production of raw materials from algae, lignocellulosic, and organic wastes.

## Introduction

The use of plastics and polymers has defined the so-called plastic age in the history line of mankind. While their properties have provided advantages in many fields from the automobile industry to the electronics, over the years, the disposal of equipment and packages has created a problem.^1,2^ Packages represent one-third of the use of plastics,^3^ and recycling has become a primary target across countries. However, the amount of plastics that has already reached the oceans has become a major concern due to the effect microplastics already has and may have in the long term on human and animal health and ecosystems.^4,5^ Taking into account the large share of plastics used for packaging, substituting them by biodegradable polymers can provide an immediate positive effect. Biodegradable polymers can be part of the larger trend of circular economy. A number of examples have already been evaluated at least at the process scale, such as polyhydroxyalkanoate (PHA),^6,7^ polyhydroxybutyrate (PHB),^8^ polylactic acid (PLA), polybutyrate (PBAT), and polyesters from adipic acid and glycerol^9^ or polymers based on starch.^10^ In addition to biodegradable polymers, it is important to focus on the synthesis of polymers from renewable resources and wastes to reduce their CO_2_ footprint and promote the principles of circular economy^11^ and the targets of the agenda 2030 of the United Nations.^12^ The waste to chemicals initiative aims at producing added value products from waste biomass,^11^ and polymers qualify within this set of products including food additives, active principles in drugs,^13,14^ and platform chemicals.^15^

For instance, the polymers presented in previous work^10^ required several raw materials including sawdust, glycerol, and starch. Sawdust is a byproduct or a residue from the forest industry, glycerol is the byproduct of the production of biodiesel,^16^ and starch is a constitutive of cereal grains or algae.^17^ Biodiesel can be produced from residues, cooking oil, and algae. In addition, algae require nutrients and a source of carbon, with CO_2_ being the one that would allow capturing it from industrial flue gases, while the synthesis of biodiesel requires an alcohol. Both can be obtained from waste.^18^ The current society generates large volumes of different wastes at industrial and residential sectors, being resources to obtain biopolymer added value products.^19−21^ Anaerobic digestion is deemed as one of the most promising technologies to manage residues with a large moisture content. The digestion results in the production of a digestate, rich in nutrients, that can be used a fertilizer as well as a biogas. Biogas, a mixture of CO_2_ and methane, contains all the building blocks required for the production of syngas, a versatile mixture.^22^ The CO_2_ that is not required for synthesis can be fed to the algae growing stage, while the syngas is employed to obtain methanol needed for the transesterification of the oil extracted from the algae. The algae starch will close the circle. Therefore, process integration can allow the sustainable production of polyols to obtain biopolymers in our attempt to substitute packaging, providing the means to reduce the use of external raw materials, improving the economics and/or the environmental impact,^23−26^ as well as developing a circular economy into the polymer production sector.

This work proposes a novel conceptual design of an integrated process for the production of biodegradable plastics from known technologies based on waste processing such as anaerobic digestion of sludge or manure, CO_2_ capture, and liquefaction of lignocellulosic materials, allowing the production of necessary intermediates for the synthesis of biopolymers such as methanol, starch, and glycerol, together with high value-added products as biodiesel. The rest of the paper is organized as follows. First, the integrated process is described. Next, the major modeling assumptions of each of the sections are presented. Subsequently, the results of the optimal operation of the facility are shown together with an economic evaluation for the two study cases: manure and sludge waste. Finally, conclusions are drawn.

## Process Description

**Figure 1:**
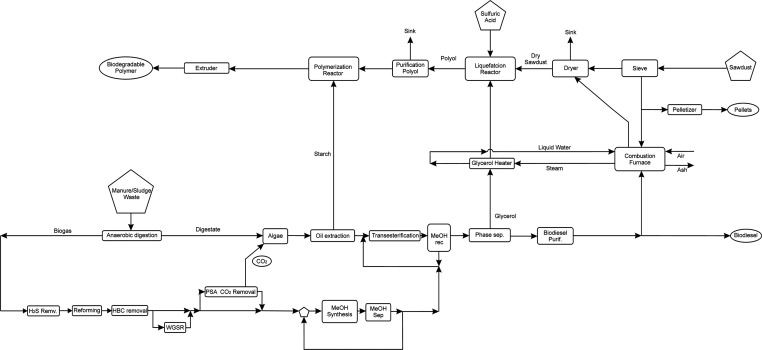
Integrated facility for biopolymer and biodiesel production from biomass wastes.

The raw materials are CO_2_, sawdust, sludge/manure, and residues from industries such as concrete, the forest or residential areas, and farms daily routine. Starting from organic wastes, they are digested anaerobically to produce biogas and digestate. CO_2_ is injected in ponds as a carbon source for the growth of algae that use digestate as a source of nutrients. Water is evaporated, and oxygen is also produced. The algae are harvested, and the oil is extracted using mechanical action and cyclohexane that is recovered via distillation, while the starch is also recovered. Transesterification of such oil requires renewable methanol that is produced via biogas reforming. Steam is added if needed. A syngas is produced whose composition is adjusted to the one required for methanol production. CO_2_ in excess is recycled to the algae growing ponds, and the syngas is used to obtain methanol. Once biodiesel is produced, the excess of methanol is recycled, and the non-polar (biodiesel) and polar (glycerol and water) phases are separated. After a preliminary drying pretreatment, the sawdust is added to the liquefaction reactor together with glycerol and sulfuric acid as the catalyst where the polyol is produced. Once the polyol has been purified by mechanical centrifugation, it is mixed with the algae starch to produce the biopolymer. The biodegradable plastic is extruded to obtain pellets useful as raw material to generate plastic-based pieces or plastic films for agricultural applications.^27^ Therefore, in this work, a conceptual design of an integrated facility from lignocellulosic and biomass waste to biodegradable plastic is presented. It includes a section for the production of biodiesel so that the main raw materials are produced internally promoting a reduction in the purchase of them. Figure 1 shows a scheme of the integrated process. The models of the different units are described in the following section.

## Modeling Issues

### Biogas Production

**Figure 2:**
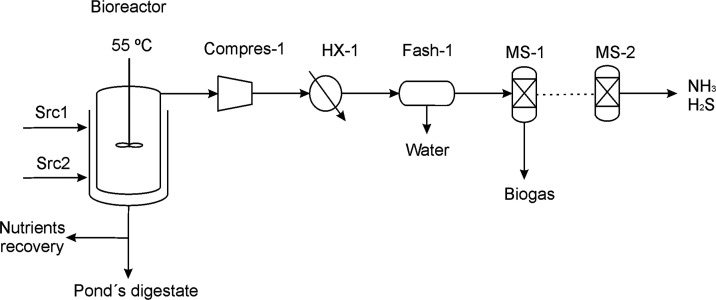
Biogas production section.

The sections of biogas generation and cleaning are shown in Figure 2. The anaerobic digester (Bioreactor) carries out a microbiological process of decomposition of manure or sludge without oxygen. The initial material (carbohydrates, lipids, and proteins) is broken down by continuous and parallel reactions such as hydrolysis, acidogenesis, acetogenesis, and methanogenesis into methane and CO_2_. Thermophilic conditions (55 °C) with a retention time of 15–20 days have been selected because this improves the growth rate of methanogenic bacteria and reduces the retention time, making a faster and more effective process.^28^ In addition to methane and CO_2_, the biogas produced is saturated with water and contains traces of other gases such as nitrogen, H_2_S, and NH_3_.^29^ Therefore, a clean-up treatment is needed before using the produced biogas.

The biogas produced is compressed and cooled down to remove water vapor by condensation. The compression stage is modeled as polytropic. Subsequently, the traces of NH_3_ and H_2_S from biogas are removed by a packed bed column that operates at 25 °C (MS-1), while a second one is undergoing regeneration (MS-2). They are composed by a mixed bed made by iron oxide (III) and zeolites to remove hydrogen sulfide and ammonia, respectively.

For the removal of H_2_S, the following chemical reaction given by eq 1 takes place^30^
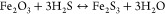
1

A removal efficiency of 100% is considered for ammonia, while sour gas removal follows the stoichiometry of the above reaction with a complete conversion of H_2_S. The bed of Fe_2_O_3_ can be regenerated with oxygen according to the following reaction, given by eq 2.^30^
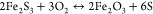
2

A fraction of the digestate produced after anaerobic digestion is sent to the ponds as nutrients for algae growth, while the rest is processed to extract the nutrients for fertilizer production.^31^

### Biogas Reforming

**Figure 3:**
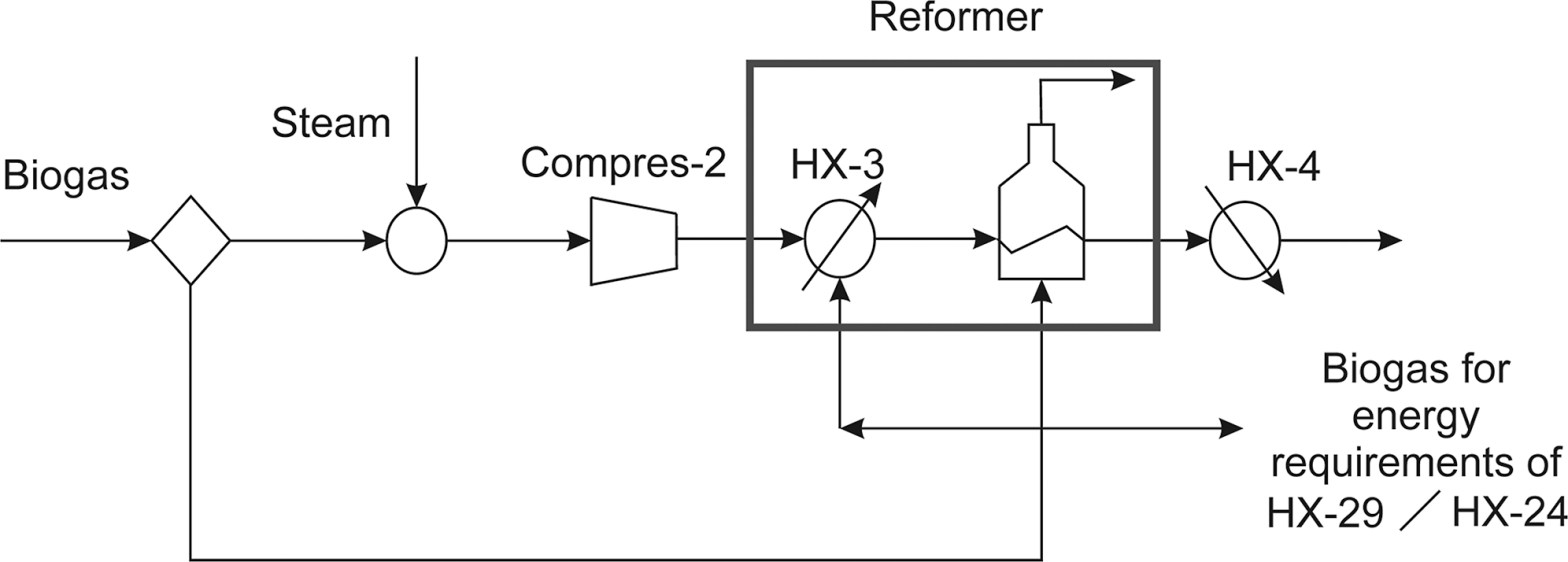
Syngas production process.

The reforming of biogas is intended to produce syngas via dry or hybrid reforming with steam. Compression and heating stages are used to adjust biogas pressure and temperature conditions for the reformer. Mass and energy balances are considered to model the heat exchangers, while the compression stage is modeled as polytropic. In spite of the endothermic nature of the reforming stage, the modeling of the reformer has been done considering an isothermal process at high temperature (800–1000 °C). To pre-heat the biogas and to maintain the operational temperature of the reformer, a fraction of the biogas is used to satisfy the energy requirements, see Figure 3. For the manure's case, additionally, the fraction of biogas is not only used to provide energy to reformer but is also employed for the energy requirement of the equipment HX-3, HX-24, and HX-29. In the case of study that uses sludge, a fraction of biogas is used in the same equipment as before. In both the reformer and the heat exchangers mentioned above, the streams are heated through the tubes of a furnace by the burning of the biogas. The fraction of biogas used as fuel is calculated using the lower heating value of the methane from biogas.

The reformer stage is based on the following chemical equilibria given by eqs 3–5. In addition, an energy balance is also formulated together with the atom balance.
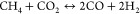
3
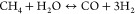
4
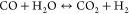
5

The syngas obtained from the reforming stage is saturated with water and contains traces of hydrocarbons. Thus, cooling and compression stages are required to remove the excess water by condensation to adapt the stream to the operating conditions of the pressure swing absorption equipment (PSA), (HBR-1). The traces of hydrocarbons are removed by a silica gel bed operating at 25 °C and 4.5 bar, while a second parallel unit is undergoing regeneration to get continuous operation (HBR-2). As mentioned before, the compression stage is modeled as a polytropic one; meanwhile, the Antoine correlation is used to calculate the remaining water within the syngas after the condensation. A pressure drop through the PSA column of 10% with regard to the feed pressure is considered.^32^

**Figure 4:**
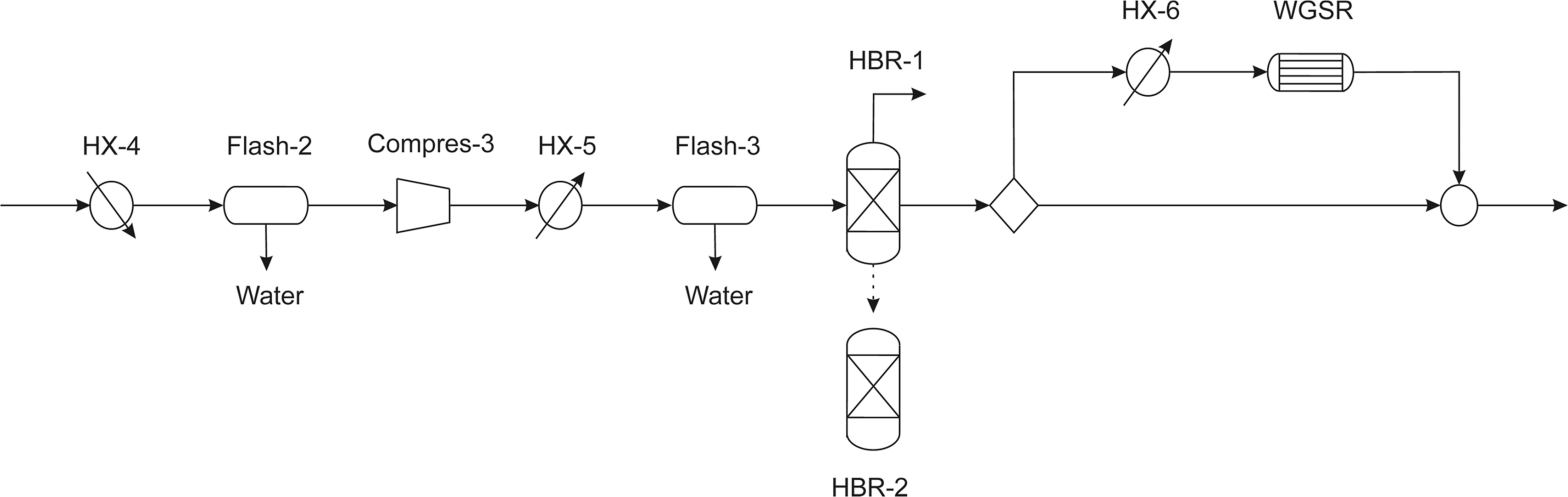
Syngas composition adjustment.

To synthesize methanol, an adjustment of the H_2_/CO ratio in the syngas is required. In this way, two parallel technologies are considered. A water gas shift reactor (WGSR) is the first technology which may be employed. It is modeled based on the equilibrium reaction given by eq 5. The second one is a bypass which mixes reformed syngas with the exit stream of the WGSR, so the percentage of syngas through the WGSR system depends on the reformeŕs performance. Figure 4 shows the process described above. More information can be found in the work of Hernández and Martín.^22^

**Figure 5:**
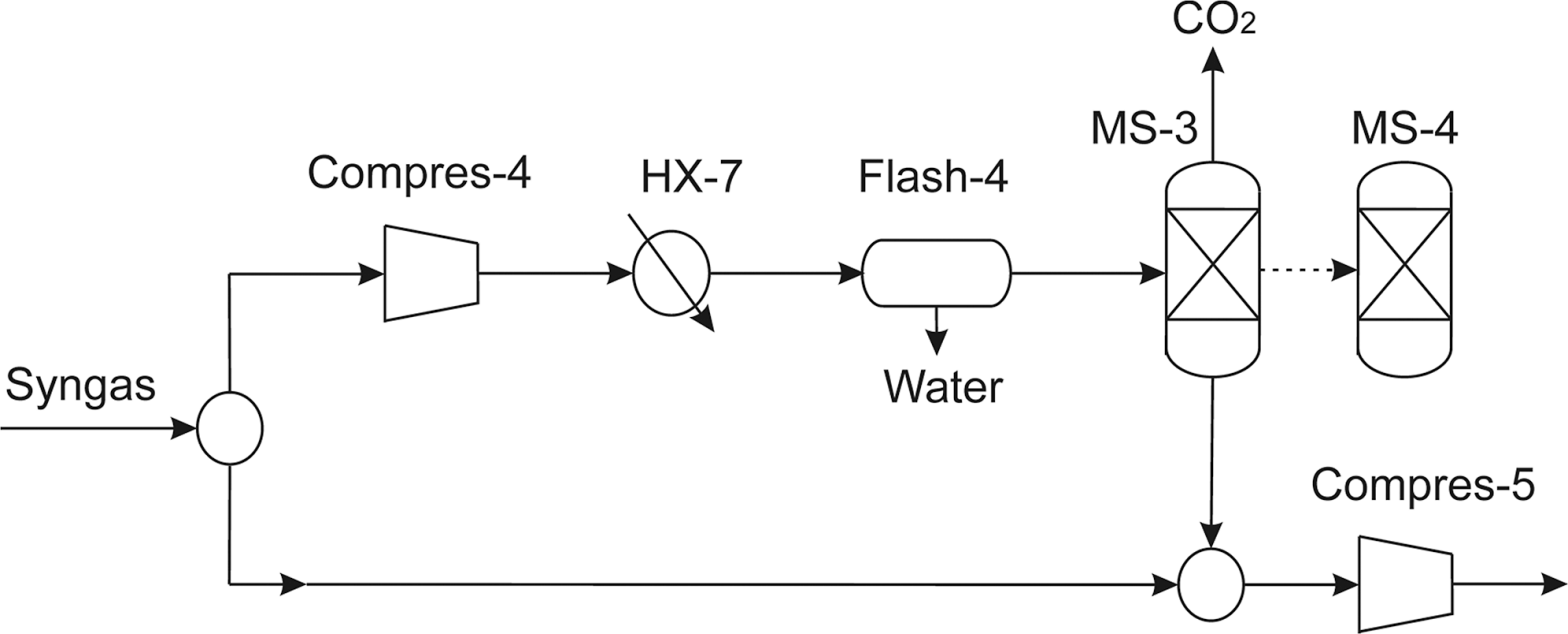
CO_2_ removal stage.

The resulting syngas after composition adjustment is subjected to a final purification stage to remove CO_2_. However, CO_2_ is essential in the synthesis of methanol. It is desirable to maintain from of 2 to 8% by volume.^33^ For this reason, two parallel and simultaneous technological alternatives are considered. The first one consists of a PSA unit (MS-3) where a syngas fraction is processed to remove the excess of CO_2_ operating at 25 °C and 4.5 bar by a Zeolite 5A or 13X bed, while a second column is undergoing regeneration (MS-4). For this purpose, a fraction of syngas is compressed and cooled down to remove the water condensed before being fed to the PSA unit, only if any amount of water is needed to be removed from the system. The efficiency of CO_2_ removal is assumed to be 95%.^34,35^ The parallel alternative to the PSA column is a bypass that sends unprocessed syngas to be mixed together with the stream of syngas from the CO_2_ removal system. Syngas’ composition adjustment is shown in Figure 5. The captured CO_2_ is fed to the algae ponds.

### Methanol Synthesis

**Figure 6:**
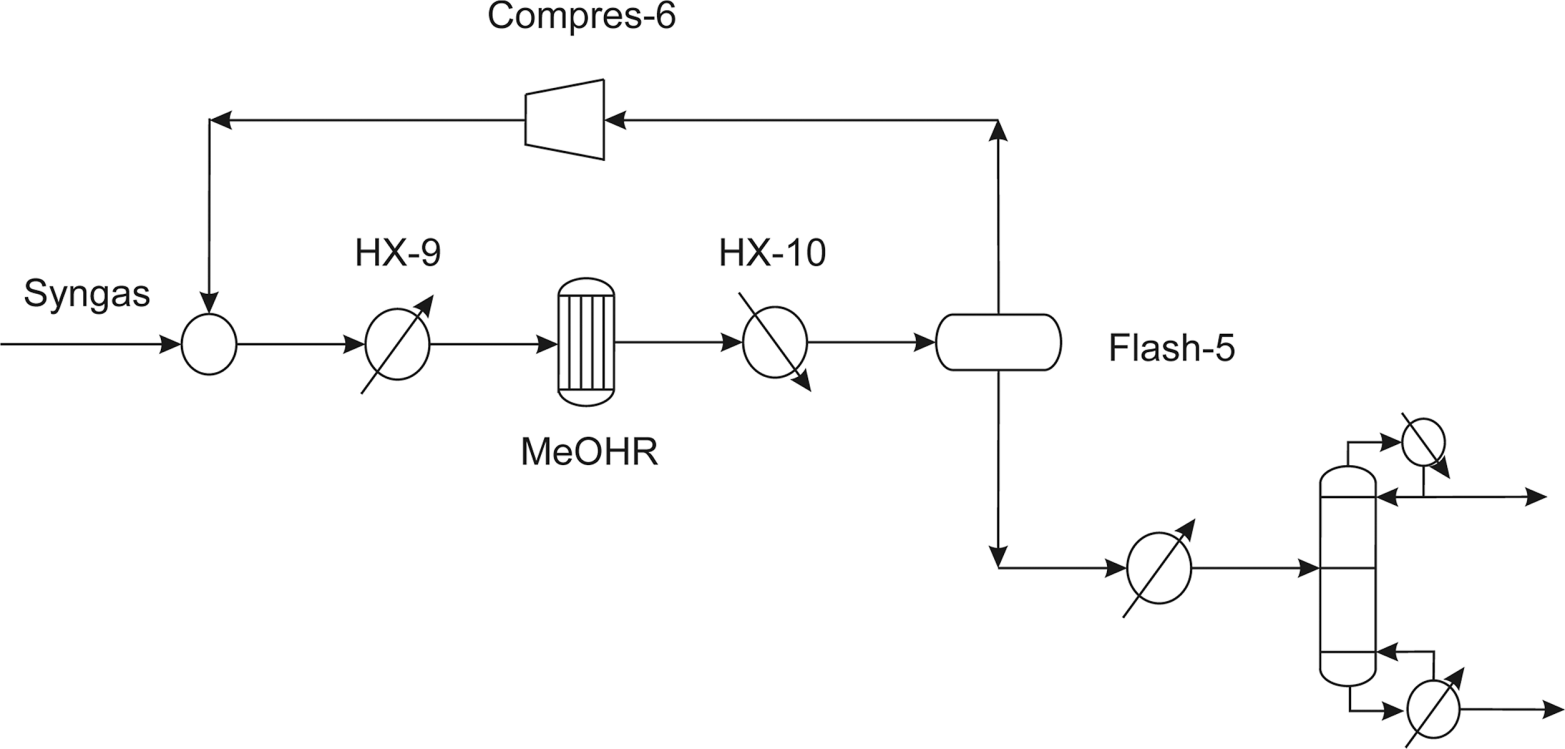
Methanol production stage.

Once the composition of the syngas has been adjusted, it is sent to the synthesis loop for the methanol production, presented in detail in Figure 6. The methanol reactor (MeOHR) employs a catalyst composed of CuO–ZnO–Al_2_O_3_ for the conversion of the syngas to methanol. The model of the synthesis reactor is based on an elementary mass balance associated with the chemical equilibria involved in the reaction process given by eqs 6 and 7.
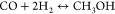
6
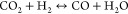
7

High pressures and low temperatures promote the production of methanol. However, the most common conditions in the reactor are 50–100 bar and a range of temperatures between 200 and 300 °C. The proper operation conditions to be satisfied by the feed of the reactor are the ratio of the syngas components given by eq 8(36,37) and a concentration of CO_2_ between 2 and 8%.^33^
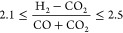
8

The outlet gases of the methanol synthesis stage are sent to a flash unit in which the unreacted syngas are recycled forward the reactoŕs feed; meanwhile, methanol is recovered. A system of molecular sieves or distillation column may be needed if further a purification of methanol is required, depending on the performance of the reactor, to remove the water produced during the synthesis such as is shown in Figure 6. A fraction of the methanol produced is employed in the transesterification reactor while the excess is sold. However, if the methanol produced out of syngas is not enough, it has to be purchased at a cost.

### Oil Production

**Figure 7:**
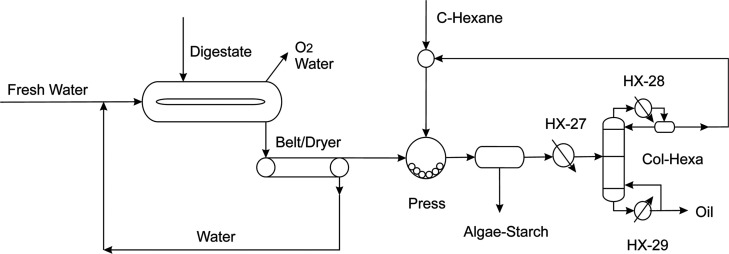
Starch and algae oil production stage.

The algae growing and oil extraction section is presented in Figure 7.

Water quality, temperature, minerals, carbon source (carbon dioxide), nutrients, and light cycle and intensity are factors which determine the algae growth. In terms of nutrients, the total nitrogen and total phosphorus are the most important factors for algae production which are provided by digestate. Algae growth (growth_algae_) is quantified based on a correlation in terms of the effect of the nutrient concentrations given by eq 9.^18^ The total phosphorus (TotP) and the total nitrogen (TotN) concentrations are given in milligrams per liter.
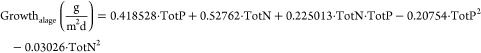
9

As the carbon source, CO_2_ is consumed. The relationship between the consumed rate of CO_2_ () and the growth rate of algae is given by eq 10.^38^
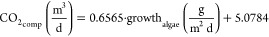
10

The water requirements for algae growth are provided from two sources. The first one is the digestate from the anaerobic digester that carries an amount of water. The second one is an extra water source that may need to be employed to achieve an algae concentration of 0.006 kg per kg of biomass.^39^ The energy requirements for the operation of the ponds is computed based on Sazdanoff's data.^38^ Once the algae growth is completed, the algae are harvested and sent to a drying stage until the moisture content is reduced to 5% employing Univenture’s design. This pretreatment stage has an energy consumption of 40 W per 500 L/h of flow.^40^ The water used is recycled to the ponds so that only a evaporation water loss to be fed again.

The pretreated algae are mixed with cyclohexane, which is used as a solvent. The mixture is processed by a mechanical press to extract the oil and the starch from algae, which is separated from the oil and cyclohexane. Finally, the oil and the solvent are separated in a vacuum distillation column (Col-Hexa). The bottoms must be below 350 °C to avoid oil decomposition. In this way, the oil recovered is sent to the transesterification reactor, while the solvent is recycled. The oil and the starch extracted represent 55 and 35% of the dry biomass, respectively, and the rest is considered as protein.^18^

### Biodiesel Synthesis

A heterogeneous catalysis method for biodiesel synthesis is selected. The advantage of using this method is the capability of employing different oil sources and promote an easier product separation. Therefore, biodiesel washing is no needed, contributing to a reduction of fresh water consumption in the process. The transesterification reaction yield (yield_trans_) is calculated by a surface response model given by eq 11.^16^ The upper and lower bounds for each factors in eq 11 are shown by Table 1.
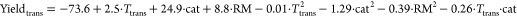
11

**Table 1:** Range of Operation of the Variables^a^

Variable	Lower bound	Upper bound
Transesterification temperature: *T*_trans_(°C)	40	60
RM: ratio methanol (mol/mol)	6	12
Percentage of catalyst: cat (%)	1	4

a Heterogeneous catalysis for biodiesel production.

**Figure 8:**
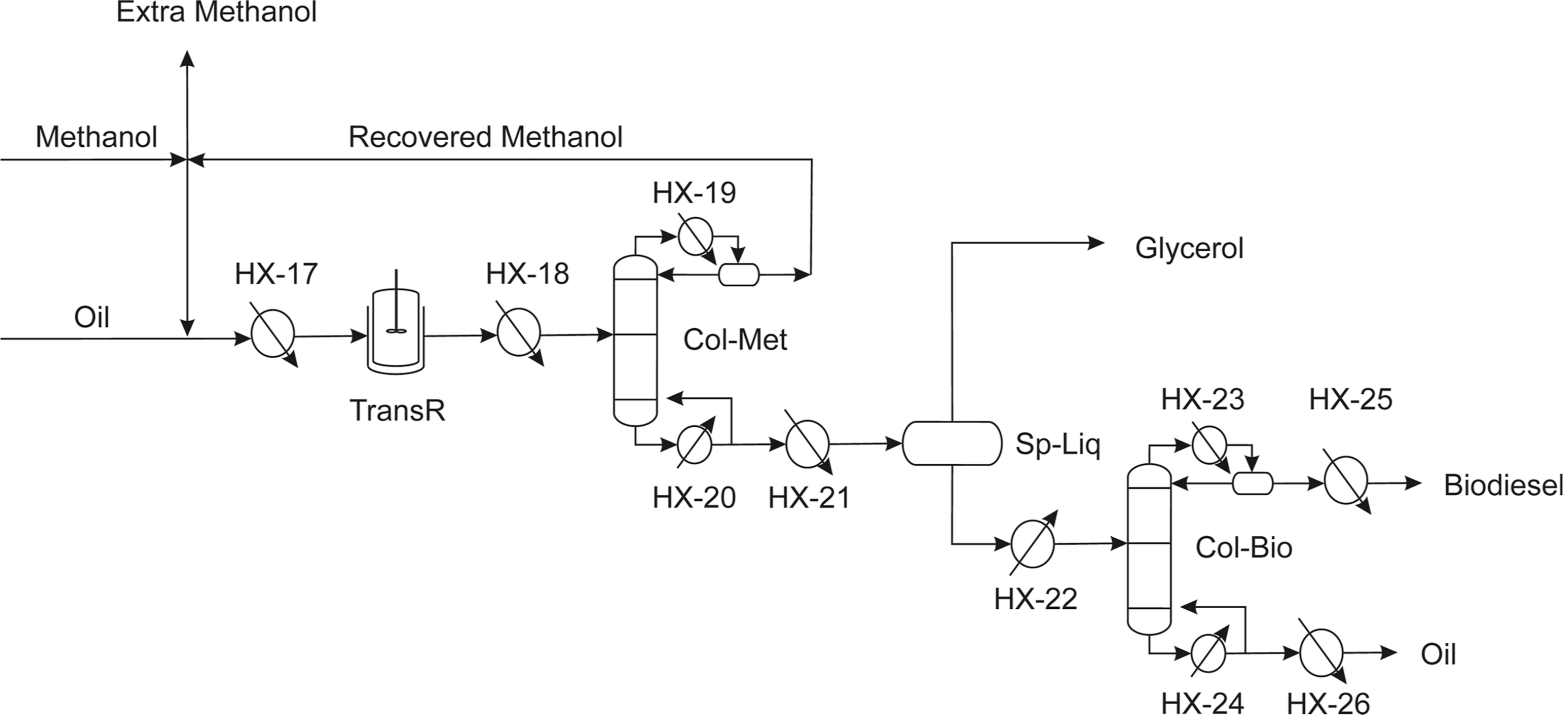
Biodiesel production section. Based on ref (41).

The unreacted methanol from the transesterification reactor (TransR) is recovered and recycled using a distillation column (Col-Met) which operates under vacuum conditions and is modeled based on shortcut methods considering the existence of a polar and non-polar liquid phases. A phase separator (Sp-Liq) is followed by vacuum distillation where the polar and non-polar phases are separated at 60 °C. To purify the biodiesel from the oil, the non-polar phase from the separator is treated in a vacuum distillation column (Col-Bio) so that a distillate above 250 °C is avoided. The synthesis and purification stage of biodiesel are shown in Figure 8.

**Figure 9:**
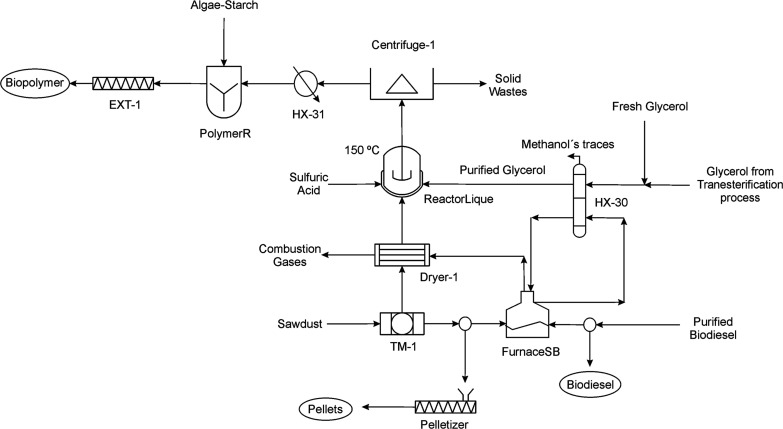
Flowsheet for the production of biopolymers based on glycerol.

A fraction of biodiesel recovered is sold, and the rest is sent to the furnace (FurnaceSB) as fuel to produce steam at 230 °C, which is employed as a heating fluid in the falling film evaporator (HX-30). An additive source of fresh glycerol based on cooking oil^16^ is fed to the process and mixed with the recovered glycerol if the last one is not enough for the liquefaction of the sawdust. The glycerol is sent to the film evaporator to heat it up slightly above the liquefaction reaction temperature, 150 °C,^10^ since when it is mixed with the other components involved in the solvolysis reaction in the liquefaction reactor (ReactorLique), a fraction of the heat is transferred to the rest of components to reach the reaction temperature. The traces of methanol present in the glycerol are removed by evaporation. The pretreatment of glycerol explained above is shown in Figure 9.

### Biopolymer Production

The biopolymer production is based on four sections.^10^ The initial stage is a pretreatment of the sawdust used as the lignocellulosic biomass source. A sieve within a vibrating screen is employed to homogenize the particle size of the sawdust between 0.3 and 0.6 mm.^42^ The total homogenized sawdust may be used in two ways. A fraction can be employed for the production of bioplastics and the rest is sent to a splitter. This stream is divided to produce pellets of sawdust and the rest is used as fuel together with biodiesel in the furnace (FurnaceSB) to produce steam, employed in the glycerols heating, see Figure 9. The combustion chamber is modeled as an adiabatic furnace where biodiesel and/or sawdust is burned with excess of air of 65%. The fractions of the total sawdust employed to produce biopolymers, pellets or to be burned in the furnace, are computed by the optimal solution of the process model. The fraction of sawdust devoted to the production of biodegradable plastic is dried in a direct contact rotatory dryer with a moisture removal yield of 90% in which the combustion gases from the furnace (FurnaceSB) are used as a drying fluid.

Liquefaction reaction of sawdust is the second stage for the biopolymer production. The dried sawdust is introduced in the liquefaction bath reactor (ReactorLique) together with glycerol and sulfuric acid as a catalyst. A glycerol-to-sawdust ratio of 5.04:1 and a sawdust-to-acid sulfuric ratio of 8.88:1^42^ are considered. The sawdust and sulfuric acid are fed at 25 °C while the glycerol is introduced at 165 °C. Before the reaction starts, these three components are placed in the bath reactor. A heat transfer is carried out from glycerol to the rest of the raw materials to reach the reaction temperature as described previously. In this way, the liquefaction reactor of the lignocellulosic biomass operates at 150 °C and atmospheric pressure during 45 min.

The yield of biomass liquefaction depends on the selected solvent.^43^ Because it is the major component of the mixture of polyols obtained, the selected solvent fixes the features of the polymer based on polyols. Glycerol breaks the biomass structure, obtaining highly reactive compounds which are recombined with biomass or glycerol, producing a mixture of polyols,^44^ being commonly used in the liquefaction of lignocellulosic wastes.^45^ The liquefaction reaction has a reaction heat of −186.72 kJ/kg;^42^ therefore, a cooling jacket is needed to remove the thermal energy produced to keep a constant temperature during the liquefaction.

A response surface model based on experimental data was developed to quantify the liquefaction conversion (Conv_lique) as a function of temperature (*T*_liquefact_) and time (*t*_liquefact_), eq 12.^42^ For the reaction conditions of 150 °C and 45 min, a conversion of 99.7 is obtained, assuming a selectivity of 100%.
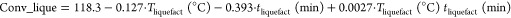
12

A third stage is needed to purify the polyol produced in the liquefaction stage. The mixture of polyols obtained from the solvolysis reactor contains traces of glycerol, sulfuric acid, water, and solid impurities from the sawdust. A centrifuge (centrifuge—1) is employed to obtain two streams. The first one is the purified polyol with traces of water, glycerol, and sulfuric acid, while a second one is the waste stream that contains the solid impurities with a fraction of 10% of the solvent, water, and catalyst fed to the centrifuge.^42^

The purified polyol is mixed with the starch that comes from the algae mechanical extraction in the polymerization reactor (PolymerR). This process is carried out at 20–30 °C, atmospheric pressure, and a residence time of 5 min.^42^ In this way, a starch-to-polyol ratio of 2.63:1 is considered, where the polyol acts as a plasticizing agent, cross-linking the starch molecules to obtain the bioplastic.^42^

In the fourth stage, the biopolymer is extruded (EXT-1) to produce pellets, an intermediate product to be used in transformation plastic techniques (e.g., injection and extrusion) for film or laminated plastic product production. Figure 9 shows the biopolymer production process.

## Solution Procedure

The objective function considers, on the one hand, the sale of biopolymer films, biodiesel, the excess of methanol if any, and sawdust́s pellets, if a fraction of the total homogenized sawdust is used for this purpose. On the other hand, the cost of the raw materials required (e.g., sawdust, sulfuric acid, and extra fresh glycerol and methanol if they are not produced in the required amounts), the cost of the power consumed by the compressors, and the rest of the equipment and the cost for steam used for certain heat exchangers are also included in the objective function. If a larger amount of methanol is required than the produced one, it is considered as a cost in the objective function. In this way, the variable associated with the excess/required methanol is defined as a free variable for the model. Eq 13 shows the objective function considered in the non-linear problem (NLP) model.
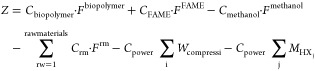
13

The constraints of the model are based on mass and energy balances, surrogate models (response surface models), and experimental data allowing to evaluate the performance of each of the units of the integrated process as it is described in the previous section, see the Supporting Information for more details. The model developed for the design of integrated facility for the production of biodegradable polymers and biodiesel is formulated as an NLP with 5690 equations and 7880 variables solved in GAMS. Ideally, a rigorous simulation of the results should be the next step after the optimization. However, in the particular scenario of the use of novel units and biomass whose properties or blocks are not defined within rigorous simulators, no advantage can be found by simulating the entire flow sheet using ASPEN or CHEMCAD. Therefore, beyond the validation of the distillation column for the separation of the hexane, a rigorous simulator is of no further use.

The non-linear problem is solved by employing a multistart optimization approach with the solver CONOPT 3.0 as the preferred one. The Windows 10 Home machine with a Intel Core i7-9 is used to achieve the probleḿs convergence. Then, the heat exchanger network is designed following Yee and Grossmann’s model.^46^ The stream matches for energy integration for the manure and sludge case studies are provided in the Supporting Information.

Finally, an economic assessment based on the factorial method proposed by Towler and Sinnott^47^ is performed. The cost of the main chemical processing equipment is determined employing specialized web pages^48,49^ as well as the correlations from previous works of Almena and Martín^50^ and Roldán-San Antonio.^10^ The costs of the anerobic digester and the falling film evaporator can be found in the section “Appendix A. Additional equipment cost correlations”^18,48^ in the Supporting Information of this work. The results have been validated at the lab or pilot plant scale. A proper scale-up to evaluate mixing issues, heat-transfer limitations, to industrial level should be carried out, but this is out of the scope of this work.

## Results

This section shows the results obtained from the optimization of the integrated bioplastics production plant model for the two wastes considered for anaerobic digestion, manure and sludge. Both the variables and the equations that make up the models of these two studies are the same, except for the composition of the waste processed in the anaerobic digester. The first study uses manure, in particular a mixture of cattle and pig slurry with a proportion of 1:1, respectively. For the second case study, sludge from wastewater treatment is considered as feed for the anaerobic digester. Table 2 shows the biogas produced per kg and the compositions associated with manure and sludge considered in each study case.

**Table 2:** Biomass Properties

	Manure's case		Sludge's case
	cattle slurry	pig slurry	wastewater
Vbiogas	0.30	0.50	0.51
wDM	0.10	0.060	0.11
wVS	0.80	0.80	0.46
wC	0.68	0.56	0.60
wN	0.013	0.026	0.027
wNorg	0.036	0.030	0.002
wP	0.0080	0.019	0.025
wK	0.10	0.053	0.014
Rcn	14.07	10.00	20.74

### Mass and Energy Balances: Manure Case of Study

The integrated facility processes 20 kt/yr of sawdust and 4732 kt/yr of manure with a weight composition of 50% cattle manure and 50% pig manure. The plant has a production capacity of 354 kt/yr of biodegradable plastic, 84 Mgal/yr of fatty acid methyl ester (FAME), and 66 kt/yr of methanol of which 53% is sold as a byproduct, a production size within the production range for algae-based biodiesel production and sawdust-based plastic production plants.^10,17^

The logistics of a farm with such a waste generation capacity is not part of this study. As a result of the anaerobic digestion, 99 kt/yr of biogas are produced with a weight composition of 38.5% methane, 47% CO_2_, and the rest H_2_S, NH_3_, and O_2_. 34% of the total biogas generated in the digester is employed to satisfy the energy requirements for the reforming process and of those heat exchangers in which the use of steam is not possible due to their high operating temperatures such as HX-3, HX-24, and HX-29. The reforming of syngas is carried out at 990 °C and 4.50 bar. However, a water gas shift reaction is not needed in order to obtain a proper H_2_/CO ratio for the synthesis of methanol. In the final stage of purification of syngas, a 95% of CO_2_ is removed, obtaining a final mass composition of 18% in CO_2_.

For the synthesis of methanol, the optimal operating conditions in the reactor are 200 °C and 50 bar, where 28% of the total product stream is recycled as a unreacted gas back to the reactor. The recovered methanol and algae oil are mixed in a molar proportion of 6.53:1, which are fed to the transesterification reactor which operates at 4 bar and 60 °C reaching a yield of 98%, producing 0.06 kg of FAME and 0.0062 kg of glycerol per kg of manure. Table 3 shows the main operating conditions for growth algae and oil–starch extraction.

**Table 3:** Main Operating Variables of Selected Units in Manure's Case study

Number of ponds	15,000
Temperature (°C)	amb
Nitrogen from digestate (mg/L)	48.30
Phosphorus from digestate (mg/L)	33.52
Mass ratio algae/cyclohexane	1:1
Mass ratio of oil produced to manure processed	0.061
Mass ratio of starch produced to manure processed	0.040

In the biopolymer production section, the raw sawdust is fully used to the polyol synthesis. Therefore, the use of the lignocellulosic waste to production of pellets is not considered by the optimal solution, obtaining a bioplastic production of 17.52 kg per kg of lignocellulosic waste. 48.86% of the glycerol employed in the liquefaction reaction comes from algae. Alternatively, to satisfy the energy requirements for the film evaporator, 0.52% of the FAME produced is used as fuel in the combustion chamber (FurnaceSB). The use of biodiesel as fuel instead of the sawdust is the best option, employing all the sawdust for biopolymer production. The heat, cooling, and power requirements in the polymer section are 2990 kJ, 1711 kJ, and 23,261 kJ per kg of processed sawdust, respectively, obtaining similar values on previous works.^10^ A summary of the operating conditions for the main equipment in the integrated process is shown in Table 4.

**Table 4:** Operating Conditions of Main Equipment in Manuree's case of study (T: TOP and B: Bottom)

Unit/op. condition	Reformer	Transesterification reactor	Oil distillation column	Methanol distillation column	FAME distillation column	Methanol synthesis reactor	Liquefaction Reactor	WGSR
*T* (°C)	990	60	*T*: 18	*T*: 41.52	*T*: 243.12	200	150	200
			B:306	B:150	B:274.72			
*P* (bar)	4.50	4	0.098	0.40	0.060	50	1	4.50
molar ratio 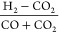						2.50		
molar ratio MeOH/oil		6.53						
biogas fuel %	19							
CO_2_ captured (kg/s)						0.25		
extra glycerol required %							51.14	

Table 5 shows the power consumption for the different stages. The integrated plant has an energy requirement of 35 and 53 MW for heating and cooling, respectively. After the energy integration of the process, a total of 112 kW are provided by steam and 40 MW are rejected by cooling water, where a consumption of 0.0058 kg of steam per kg of biopolymer produced is required. Furthermore, the process captures 2.47 kg of CO_2_ per kg of biopolymer produced, where 0.90% comes from CO_2_ capture for syngas purification (for methanol synthesis), while the rest is captured from industrial flue gas.

**Table 5:** Power Consumption of the Units Involved in Manure’s case of study

Unit	Power (kW)
Algae production ponds	1500
Harvesting belt	838
Mechanical press	3813
Biogas production	564
Reforming	0
Syngas purification	54
Methanol synthesis	1873
Polymer section	15,120

### Mass and Energy Balances: Sludge Case of Study

A total of 4653 kt/yr of wastewater is processed together with 20 kt/yr of sawdust with a production plant capacity of 354 kt/yr of biodegradable plastic, 84 Mgal/yr of FAME, and 69 kt/yr of methanol, of which 55% is sold as a byproduct. After anaerobic digestion, 104 kt/yr of biogas is produced with a weight composition of 38.5% methane, 47% CO_2_, and the rest traces of H_2_S, NH_3_, and O_2_. To satisfy the energy requirements for the reforming process and those heat exchangers which have high operating temperatures such as HX-3, HX-24, and HX-29, a 33% of the total biogas from anaerobic digester is employed as fuel. The rest of the biogas is sent to the reformer, which operates at 4.50 bar and 920 °C. As well as in the case of manure, a water gas shift reaction is not needed in order to obtain a proper H_2_/CO ratio for the synthesis of methanol. In the final stage of purification of the syngas, a final mass composition of 18.30% in CO_2_ is obtained.

In the methanol synthesis section, the reactor operates at 200 °C and 50 bar, where 28.60% of the total product stream is recycled as a unreacted gas back to the reactor. Once the methanol has been recovered, it is mixed together with the algae oil in a molar proportion of 6.53:1 before being fed to the transesterification reactor. The yield in the biodiesel synthesis is 98% under at 4 bar and 60 °C, with a production of 0.061 kg of FAME and 0.0063 of glycerol per kg of sludge. Table 6 shows the main operating condition for growth algae and oil–starch extraction.

**Table 6:** Main Operating Variables of Selected Units in Sludge's case of study

Number of ponds	15,000
Temperature (°C)	amb
Nitrogen from digestate (mg/L)	102.98
Phosphorus from digestate (mg/L)	95.10
Mass ratio algae/cyclohexane	1:1
Mass ratio of oil produced to sludge processed	0.062
Mass ratio of starch produced to sludge processed	0.041

The optimal solution shows that the sawdust is fully used for the synthesis of polyol in the biopolymer production section, obtaining 17.52 kg of bioplastic per kg of lignocellulosic waste. 51.14% of the glycerol employed in the liquefaction reaction comes from the transesterification of cooked oil. To satisfy the energy requirements for the film evaporator used for heating the glycerol fed to the liquefaction reactor, 0.52% of the biodiesel produced is fed as fuel in the furnace (FurnaceSB). The heat, cooling, and power requirements in the polymer section are 2990 kJ, 1711 kJ, and 23,261 kJ per kg of the raw sawdust processed, respectively. A summary of the operating conditions for the main equipment in the integrated process for sludge waste are shown in Table 7.

**Table 7:** Operating Conditions of Main Equipment in Sludge'´s case of study (T: TOP and B: Bottom)

Unit/op. condition	Reformer	Transesterification reactor	Oil distillation column	Methanol distillation column	FAME distillation column	Methanol synthesis reactor	Liquefaction reactor	WGSR
*T* (°C)	920	60	*T*: 18	*T*: 41.52	*T*: 243.12	200	150	200
			B:306	B:150	B:274.72			
*P* (bar)	4.50	4	0.098	0.40	0.060	50	1	4.50
molar ratio 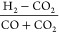								
molar ratio MeOH/Oil		6.53						
biogas fuel %	19							
CO_2_ captured (kg/s)						0.29		
extra glycerol required %							51.14	

Power consumption for the different stages is shown in Table 8. The integrated plant requires 68 and 47 MW for heating and cooling, respectively. Once the energy integration is done, a total of 49 MW are provided by steam and 33 MW are removed by cooling water, employing a 2.56 kg of steam per kg of biopolymer. Furthermore, the process captures 2.47 kg of CO_2_ per kg of the biopolymer produced, where 1% comes from CO_2_ capture for syngas purification (for methanol synthesis), while the rest is captured from industrial flue gas.

**Table 8:** Power Consumption of the Units Involved in Sludge's case of study

Unit	Power (kW)
Algae production ponds	1500
Harvesting belt	838
Mechanical press	3813
Biogas production	604
Reforming	0
Syngas purification	57
Methanol synthesis	1961
Polymer section	15,120

### Economic Evaluation

**Figure 10:**
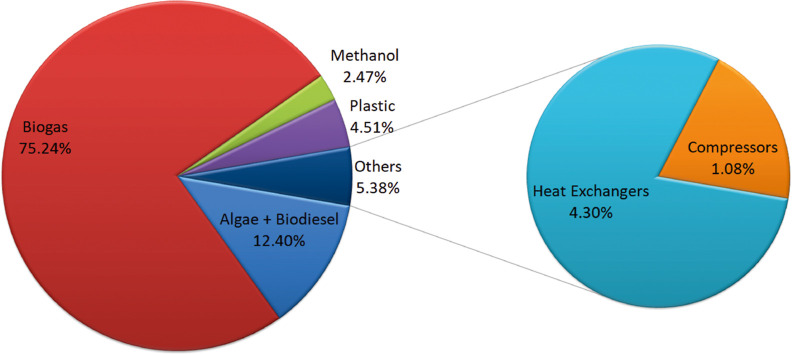
Cost distribution for main sections’ process for manure's case of study.

**Figure 11:**
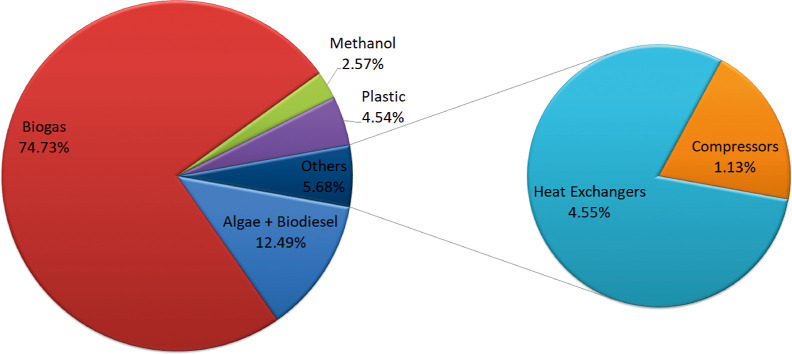
Cost distribution for main sections’ process for sludge's case of study.

The investment capital required for the facilities associated with the two cases proposed in this work is estimated considering that equipment cost, equipment erection, instrumentation, and piping which represent 26.5, 12, 4, and 12% of the fixed capital. Assuming 5% of fixed capital as the working capital, the total investment capital adds up to 717 and 712 M$ for the processes using manure and slugde, respectively. The distribution costs of the main section of the processes are shown in Figures 10 and 11, for manure and sludge based one respectively. The biogas section, consisting of the anaerobic digester and biogas clean up, is the major item for the total investment cost, representing around 75% for both case of study, showing that the anaerobic digester is the unit with the largest impact on the equipment cost item. Biodiesel and algae production section represents 12%, higher than the methanol section around 2–3% of the equipment cost in both cases. Biodiesel–algae section integrates algae growth and harvesting and the transesterification synthesis and purification of the FAME. The methanol section involves biogas reforming as well as syngas purification. The biopolymer production section includes pretreatment of sawdust, synthesis of polyol and polymerization with a similar value of around 4.5% of the equipment cost for both cases.

**Figure 12:**
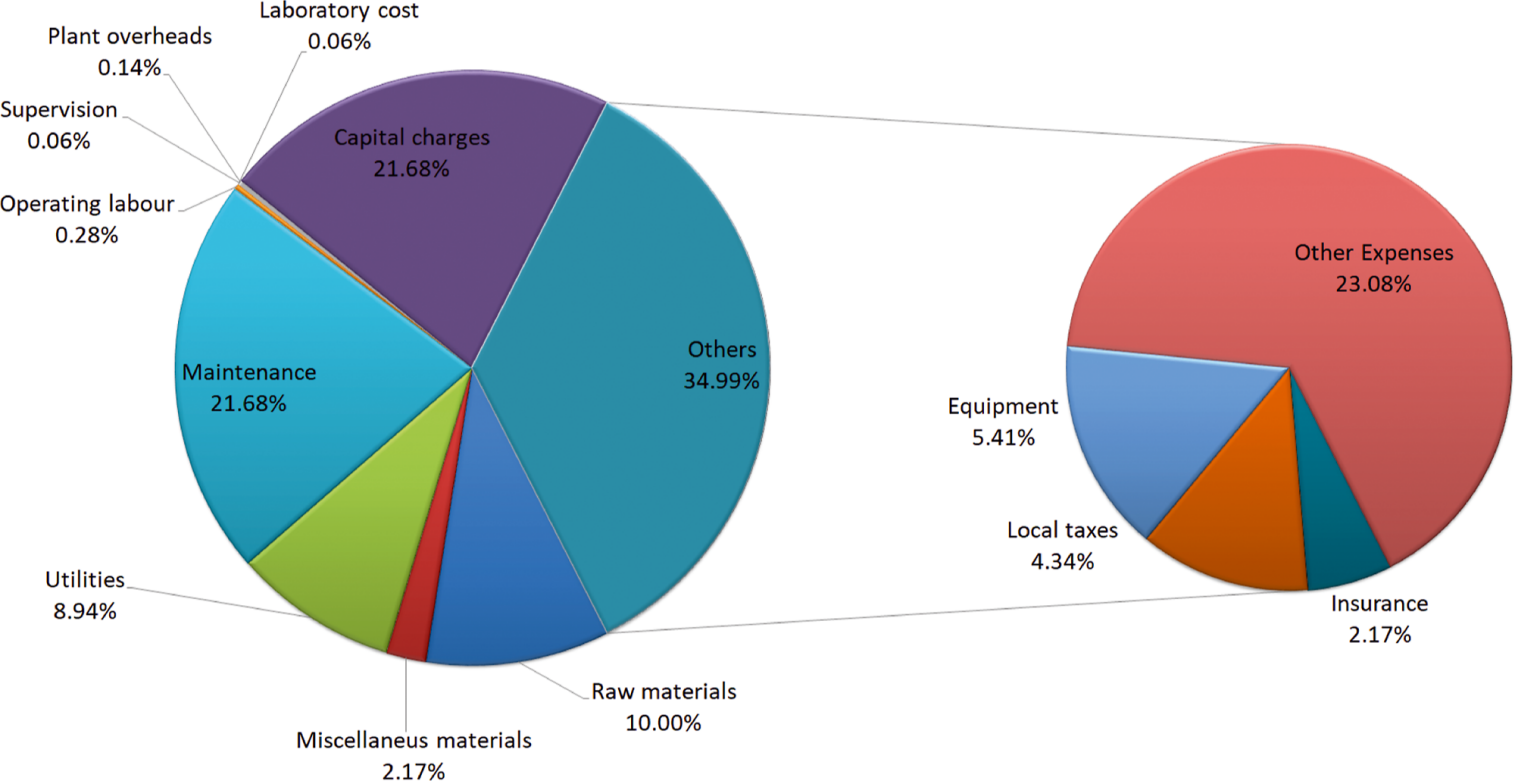
Annual distribution costs for manure process.

**Figure 13:**
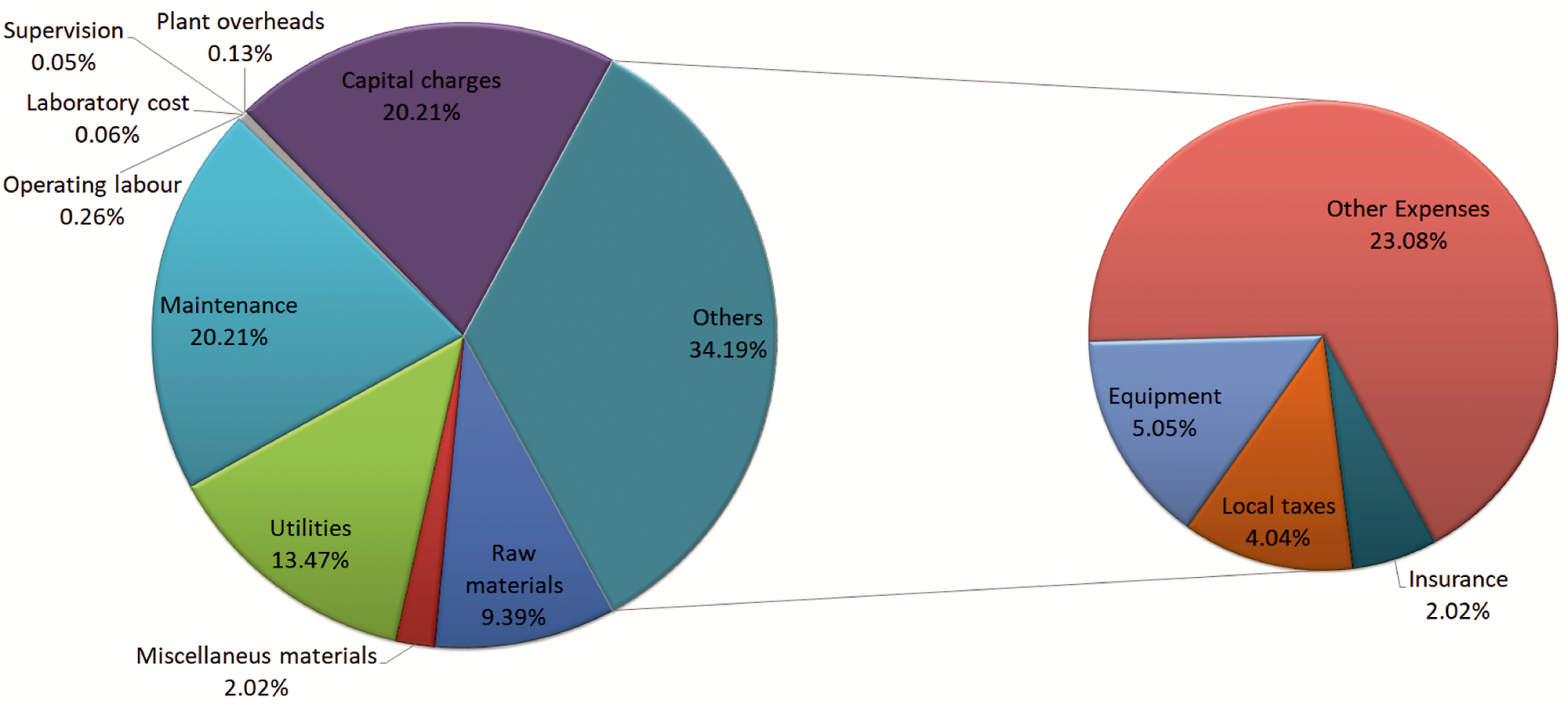
Annual distribution costs for sludge process.

The annual production cost estimation includes raw materials, utilities, equipment maintenance, operating labor, and equipment amortization as main items which add up to 315 M$/yr for the manure based process and 336 M$/yr for the sludge based one. Figures 12 and 13 show the distribution of annual production costs for both cases of study. However, due to the excess of methanol and the FAME produced, credits of 511 and 512 M$/yr are obtained for the manure- and sludge-based processes, respectively. Note that the sale of these byproducts exceeds the annual production costs so that the unit cost for bioplastic production is zero. To estimate the cost of the biopolymer, the credits from the sale of biodiesel and methanol are not included to be compared with non-integrated facilities from the previous work of Roldán-San Antonio.^10^ Thus, the unity production cost for the biopolymer when manure is used reaches a value of 0.89, while a 0.95 $/kg of biopolymer produced is when sludge is the source of nutrients. Due to the high costs involved in the investment in an anaerobic digester that processes such a large amount of waste, the amortization and maintenance costs of the digestion section are higher, leading to an increase in the annual cost and thus the unit cost of biopolymer production with respect to the stand-alone facility.^10^ However, the unity cost obtained is competitive compared with the average sale cost of plastic films in the market, around 1.825 $/kg.^51^ The cost estimation is assumed to have a 30% error of approximation.

It is important to highlight that the reason why the unity production cost of the biopolymer for sludgés process is higher than the manure based one. The higher consumption of steam (utilities item) to satisfy the energy requirements during the endothermic anaerobic digestion process of the wastewater is behind that difference, unlike the anaerobic digestion of manure which is exothermic due to its composition based on carbohydrates.^52^ Note that a larger equipment in the methanol and biodiesel sections is needed because more biogas is produced in the sludge based process, but the amortization cost is lower than the equivalent in the manure based process since the total waste processed in the anaerobic digester is lower, reducing the cost of this unit.

With regard to the profits from the sale of byproducts, the sludge based process generates 0.20% higher profit than the manure's one. The reason is that in the sludge process, a higher production of biogas is obtained so that the production of methanol is also higher. Given that the optimal oil-to-methanol ratio is the same for both cases of study, the production of FAME is the same, but the surplus of methanol to be sold as a byproduct is higher in the case of wastewater. Finally, in both cases, the integrated plant produces 2.46 kg of glycerol per kg of sawdust processed. Since 5.04 kg of glycerol per kg sawdust is required in the liquefaction reaction, it is concluded that the solvent production is limited by the algae harvest, requiring an extra glycerol from cooking oil.

In this way, both types of wastes give similar operation conditions and economic assessment. However, the use of manure promotes a reduction in the unity production cost of the polymer with a lower profits for the sale of byproducts, while the sludge process shows an increase in the sales profit with a higher unity production cost.

## Conclusions

This work presents a conceptual design and technoeconomic evaluation of an integrated facility which involves the production of biodegradable plastic and biodiesel from sawdust, CO_2_, and biomass wastes (manure and sludge). The facility produces all intermediate materials required such as methanol from biogas, oil, starch, and glycerol from the algae oil transesterification process. In this way, this process can be considered as a sustainable alternative for waste processing to produce added-value products such as biopolymers and FAME, promoting circular economy around manure or sludge and sawdust. A large NLP is formulated to optimize the operating conditions at the main equipment such as biogas reformer, methanol, biodiesel, and biopolymer synthesis.

The optimal solution shows that the sawdust is fully used as raw material for the production of biopolymer instead of being used as a fuel or for pellet production in both cases of study (manure and sludge), promoting a technological substitution of petroleum-based plastics.^53,54^ An investment capital of 717 and 712 M$ for the manure and sludge cases of studies are required, respectively, showing a high economic entry barrier because high investment is needed due to the anaerobic digestion involved in the process. However, it significantly reduces the cost of the raw materials due to the integrated plant design with a unity production cost of the bioplastic of 0.89 and 0.96 $/kg for manurés and sludgés processes respectively.

## Supporting Information Available

Models for the units involved in the process flow sheet and heat exchanger network design and description of model accuracy (PDF)

## Nomenclature

cat: percentage of the catalyst (percentage units)
*C*_biopolymer_: selling price of the biopolymer ($/kg)
*C*_FAME_: selling price of FAME ($/kg)
*C*_Methanol_: selling price of methanol ($/kg)
CO_2 comp_: consumed rate of CO_2_ in ponds ()
Conv_lique: liquefaction conversion (percentage units)
*C*_power_: cost of electricity ($/kW)
*C*_rm_: cost of raw materials ($/kg)
*C*_steam_: dost of steam ($/kg)
FAME: fatty acid methyl ester
*F*^biopolymer^: biopolymer sold (kg/s)
*F*^FAME^: FAME sold (kg/s)
*F*^methanol^: methanol sold (kg/s)
*F*^rm^: flow of raw materials required (kg/s)
growth_algae_: algae growth
LHV: lower heating value
*M*_HX/j_: Mmss of steam required in heat exchanger *j* (kg/s)
NLP: non-linear problem
*P*: pressure (bar)
PBAT: polybutyrate
PHA: polyhydroxyalkanoate
PHB: polyhydroxybutyrate
PLA: polylactic acid
ratio methanol: ratio methanol to oil in transesterification reaction (mol/mol)
*R*_CN/k_: mass ratio between carbon and nitrogen in the biomass
RM: ratio methanol (mol/mol)
rm: raw materials considered = {sawdust, sulfuric acid, glycerol based on oil cooked, extra methanol}
*T*: temperature (°C)
*T*_liquefact_: liquefaction temperature (°C)
*t*_liquefact_: liquefaction time (min)
TotN: nitrogen concentration in digestate (mg/L)
TotP: phosphorus concentration in digestate (mg/L)
*T*_trans_: transesterification temperature (°C)
*V*_biogas/k_: biogas volume produced per unit of volatile solids (VS) (m^3^_biogas_/kg_VS/k_)
*w*_C/k_: dry mass fraction of C in dry biomass (kg_C/k_/kg_DM/k_)
*W*_compress/i_: power consumption of each compress *i* (kW)
*w*_DM/k_: dry mass fraction in the biomass (kg_DM/k_/kg)
*w*_K/k_: dry mass fraction of K nitrogen in dry biomass (kg_K/k_/kg_DM/k_)
*w*_N/k_: dry mass fraction of nitrogen in dry biomass (kg_N/k_/kg_DM/k_)
*w*_Norg/k_: dry mass fraction of organic nitrogen in dry biomass (kg_Norg/k_/kg_DM/k_)
*w*_P/k_: dry mass fraction of P in dry biomass (kg_P/k_/kg_DM/k_)
*w*_VS/k_: dry mass fraction of volatile solids out of the dry biomass (kg_VS/k_/kg_DM/k_)
yield_trans_: transesterification reaction yield (percentage units)

## Compounds

CO_2_: carbon dioxide
H_2_S: hydrogen sulfide
NH_3_: ammonia
Fe_2_O_3_: iron oxide (III)
Fe_2_S_3_: iron Sulfide (III)
H_2_O: hydrogen oxide
O_2_: oxygen
S: sulfur
CH_4_: methane
CO: carbon monoxide
H_2_: hydrogen
CuO: copper oxide (II)
ZnO: zinc oxide
Al_2_O_3_: aluminum oxide
CH_3_OH: methanol

## Units

bioreactor: anaerobic digester
MS-(unit): packed bed column
HX-(unit): heat exchangers
HBR-(unit): hydrocarbon removers
WGSR: water gas shift reactor
PSA: pressure swing absorption
MeOHR: methanol reactor
Col-Hexa: vacuum distillation column for cyclohexane recovery
TransR: transesterification reactor
Col-Met: distillation column for methanol recovery
Sp-Liq: phases separator
Col-Bio: vacuum distillation column for biodiesel recovery
FurnaceSB: combustion chamber
ReactorLique: liquefaction reactor
centrifuge-(unit): centrifuge
PolymerR: polymerization reactor
EXT-(unit): biopolymer extruder
