# Supplementary material for: Optimal Integrated Plant for Biodegradable Polymer Production
Source: ACS Sustain Chem Eng. 2023 Feb 2;11(6):2172–85. doi: 10.1021/acssuschemeng.2c05356 (PMC9930116; doi:10.1021/acssuschemeng.2c05356)
Supplement: Supplementary file 1 — sc2c05356_si_001.pdf [file sc2c05356_si_001.pdf]

Optimal integrated plant for biodegradable polymers production

José E. Roldán-San Antonio[a], Mariano Martín[a1]

[a]Department of Chemical Engineering, University of Salamanca, Plaza Caídos 1-5, 37008, Salamanca, Spain

Number of pages: 26
Number of tables: 9
Number of figures: 2

[1] M. Martín mariano.m3@usal.es

Anaerobic Digestion

The yield to biogas from waste depends on the type of raw material added to the anaerobic digester as well as the temperature, digestion systems, retention time [1,2]. A mass balance is performed considering the composition of the different wastes sources in order to compute the final biogas composition [3]. The composition of the biogas resulting from the digestion is limited by typical literature limits [2], assuming that it is saturated with water. The determination of the composition is carried out on the basis of eqs (1-7). The lower and upper limits for the generation of biogas are given by eq (7) [4]:

$$MW_{dry-biogas} = \sum_{a'} Y_{a'/biogas-dry} MW_{a'}$$

$$\begin{gathered} 0.7 \le Y_{CH4} \le 0.5 \\ 0.3 \le Y_{CO2} \le 0.5 \\ 0.02 \le Y_{N2} \le 0.06 \\ 0.005 \le Y_{O2} \le 0.16 \\ Y_{H2S} \le 0.002 \\ 9\cdot 10^{-5} \le Y_{NH3} \le 1\cdot 10^{-4} \end{gathered} \quad (1)$$

$$y_{biogas} = \frac{MW_{H_2O}}{MW_{biogas-dry}} \frac{Pv(T)}{P - Pv(T)} \quad (2)$$

$$F_{biogas} = \rho_{biogas} \left[w'_{SV/cattle}\, w_{MS/cattle} F_{cattle} \cdot V_{biogas/cattle} + w'_{SV/pork}\, w_{MS/pork} F_{pork} \cdot V_{biogas/pork} \right] \quad (3)$$

$$fc(H_2O)_{biogas} = y_{biogas} \cdot \sum_{a'} fc(a')_{biogas} \quad (4)$$

$$fc_{(a',Bioreactor,Compres1)} / MW_{a'} = \frac{Y_{a'/biogas-dry}}{MW_{biogas-dry}} (F_{(Bioreactor,Compres1)} - fc_{(H_2O,Bioreactor,Compres1)}) \quad (5)$$

$$MW_{biogas} \sum_a \frac{x_{a/biogas}}{MW_a} = \sum_a x_{a/biogas} \quad (6)$$

$$\begin{gathered} 0.20 \le V_{biogasbiomass} \le 0.50 \\ 0.10 \le w_{MSBiomass} \le 0.20 \\ 0.50 \le w_{VSBiomass} \le 0.80 \end{gathered} \quad (7)$$

The composition of the digestate from the digestion is determined by a mass balance [2]. The typical compositions of the biomass for the three waste types are bounded in function of previous works [5,6]. The digestate composition is computed as follow eqs (8)-(19):

$$w'_{C/k} = R_{C-N/k}\left(w'_{Norg/k} + w'_{Nam/k}\right) \quad (8)$$

$$\begin{gathered} 3 \leq R_{C-N/CS} \leq 20 \\ 0.005 \leq w'_{Nam/CS} \leq 0.047 \\ 0.005 \leq w'_{Norg/CS} \leq 0.036 \\ 0.008 \leq w'_{P/CS} \leq 0.013 \\ 0.033 \leq w'_{K/CS} \leq 0.1 \end{gathered} \quad (9)$$

$$\begin{gathered} 3 \leq R_{C-N/PS} \leq 10 \\ 0.005 \leq w'_{Nam/PS} \leq 0.095 \\ 0.005 \leq w'_{Norg/PS} \leq 0.030 \\ 0.019 \leq w'_{P/PS} \leq 0.022 \\ 0.039 \leq w'_{K/PS} \leq 0.083 \end{gathered} \quad (10)$$

$$\begin{gathered} 6 \leq R_{C-N/PS} \leq 24 \\ 0.024 \leq w'_{Nam/Sludge} \leq 0.32 \\ 0.001 \leq w'_{Norg/\ Sludge} \leq 0.002 \\ 0.009 \leq w'_{P/\ Sludge} \leq 0.063 \\ 0.008 \leq w'_{K/\ Sludge} \leq 0.015 \end{gathered} \quad (11)$$

$$w_{C/Biomass} + w_{Norg/Biomass} + w_{Nam/Biomass} + w_{P/Biomass} + w_{K/Biomass} + w_{Rest/Biomass} = 1 \quad (12)$$

$$\begin{aligned} fc(C)_{digestate} &= w'_{C/Biomass} \cdot w_{MS/Biomass} \cdot F_{Biomass} - \\ & fc(CH_4)\frac{MW_C}{MW_{CH_4}} - fc(CO_2)\frac{MW_C}{MW_{CO_2}} \end{aligned} \quad (13)$$

$$fc(Norg)_{digestate} = w'_{No/Biomass} \cdot w_{MS/Biomass} \cdot F_{Biomass} - fc(N_2)\frac{MW_N}{MW_{N_2}} \quad (14)$$

$$fc(N)_{digestate} = w'_{N/Biomass} \cdot w_{MS/Biomass} \cdot F_{Biomass} - fc(NH_3)\frac{MW_N}{MW_{NH_3}} \quad (15)$$

$$fc(P)_{digestate} = w'_{P/Biomass} \cdot w_{MS/Biomass} \cdot F_{Biomass} \quad (16)$$

$$fc(K)_{digestate} = w'_{K/Biomass} \cdot w_{MS/Biomass} \cdot F_{Biomass} \quad (17)$$

$$\begin{aligned} &fc(\mathrm{Re}\,st)_{digestate} = w'_{rest/Waste} \cdot w_{MS/Waste} \cdot F_{Waste} - \\ &fc(CH_4)_{biogas}\frac{4 \cdot MW_H}{MW_{CH_4}} - fc(CO_2)_{biogas}\frac{2 \cdot MW_O}{MW_{CO_2}} - fc(NH_3)_{biogas}\frac{3 \cdot MW_H}{MW_{NH_3}} - \\ &fc(H_2S)_{biogas} - fc(O_2)_{biogas} \end{aligned} \quad (18)$$

$$fc(H_2O)_{digestate} = (1 - w_{MS/Biomass}) \cdot F_{Biomass} - fc(H_2O)_{biogas} \quad (19)$$

The energy balance to the digester is as follows:

$$\begin{aligned} &Q_{digestor} = \Delta H_{reaction} - Fcp(T_{digestor} - T_{in}) \\ &\Delta H_{reaction} = \sum_{prod} \Delta H_{combus} - \sum_{reac\tan ts} \Delta H_{combus} \end{aligned} \quad (20)$$

Biogas Reforming

The model of the biogas reformer is based on the chemical equilibria given by eqs (21) - (23):

$$CH_4 + CO_2 \leftrightarrow 2CO + 2H_2 \quad (21)$$

$$CH_4 + H_2O \leftrightarrow CO + 3H_2 \quad (22)$$

$$CO_{(g)} + H_2O_{(g)} \leftrightarrow CO_{2(g)} + H_{2(g)} \quad (23)$$

The reactor model is based on atomic balances as is shown by eq (24). The equilibrium constants are given by eqs (25-27) [7,8]:

$$\begin{aligned} &mol_{CH_4} + mol_{CO_2} + mol_{CO}\Big|_{in} = mol_{CH_4} + mol_{CO} + mol_{CO_2}\Big|_{out} \\ &4 \cdot mol_{CH_4} + 2 \cdot mol_{H_2O}\Big|_{in} = 4 \cdot mol_{CH_4} + 2 \cdot mol_{H_2} + 2 \cdot mol_{H_2O}\Big|_{out} \\ &2 \cdot mol_{CO_2} + mol_{CO} + mol_{H_2O}\Big|_{in} = mol_{H_2O} + mol_{CO} + 2 \cdot mol_{CO_2}\Big|_{out} \end{aligned} \quad (24)$$

$$kp = e^{[31.447-\frac{29580}{T}]} = \frac{P_{CO} \cdot P_{H_2}}{P_{CH_4} \cdot P_{CO_2}} \quad (25)$$

$$kp = 10^{[-\frac{11650}{T}+13,076]} = \frac{P_{CO} \cdot P_{H_2}^3}{P_{CH_4} \cdot P_{H_2O}} \quad (26)$$

$$kp = 10^{\left[\frac{1910}{T}-1,784\right]} = \frac{P_{CO_2} \cdot P_{H_2}}{P_{CO} \cdot P_{H_2O}} \quad (27)$$

The energy requirements are computed based on energy balances as follow eqs (28-31):

$$Q_{prod} = \sum_i fc_{(i,\text{Reformer},Mix2)} \cdot (\Delta H_f + \int_{T_{ref}}^{T_{out}} CpdT) \quad (28)$$

$$Q_{reac} = \sum_{i=in} fc_{(i,\text{Reformer},Mix2)} \cdot (\Delta H_f + \int_{T_{ref}}^{T_{in}} CpdT) \quad (29)$$

$$\text{Q(Methane reformer)} = (Q_{prod} - Q_{reac}) \quad (30)$$

$$m_{CH_4}^{extra} \cdot \text{LHV}_{gas} = \text{Q(Biogas reformer)} + \text{Q(HX24)} + \text{Q(HX29)} + \text{Q(HX3)} \quad (31)$$

Methanol synthesis

The methanol synthesis reactor is based on the following equilibria:

$$\begin{aligned} CO + 2H_2 &\leftrightarrow CH_3OH \\ CO_2 + H_2 &\leftrightarrow CO + H_2O \end{aligned} \quad (32)$$

The reactor is modeled employing the eqs (33-37) based on atomic balances as follows:

$$\begin{aligned} 2 \cdot n_{H_2} + 2 \cdot n_{H_2O} \Big|_{in} - \left(2 \cdot n_{H_2} + 2 \cdot n_{H_2O} + 4 \cdot n_{CH_3OH}\right)\Big|_{out} &= 0; \\ n_{CO} + n_{CO_2} \Big|_{in} - \left(n_{CO} + n_{CO_2} + n_{CH_3OH}\right)\Big|_{out} &= 0; \end{aligned} \quad (33)$$

$$n_{CO} + 2\cdot n_{CO_2} + n_{H_2O}\Big|_{in} - \left(n_{CO} + 2\cdot n_{CO_2} + n_{H_2O} + n_{CH_3OH}\right)\Big|_{out} = 0;$$

$$\frac{\left[P_{CH_3OH}\right]}{\left[P_{CO}\right]\left[P_{H_2}\right]^2} = 10^{\left[\frac{3971}{T} - 7.492 LogT + 1.77x10^{-3}T - 3.11x10^{-8}T^2 + 9.218\right]} \quad (34)$$

$$\frac{\left[P_{CO}\right]\left[P_{H_2O}\right]}{\left[P_{CO_2}\right]\left[P_{H_2}\right]} = Exp\left[13.148 - \frac{5639.5}{T} - 1.077\ln T - 5.44x10^{-4}T + 1.125x10^{-7}T^2 + \frac{49170}{T^2}\right] \quad (35)$$

$$1.75 \le \frac{H_2}{CO} \le 3 \quad (36)$$

$$1.5 \le \frac{H_2 - CO_2}{CO + CO_2} \le 2.5 \quad (37)$$

Oil and Biodiesel production

Algae production is based on digestate from the anaerobic digestion. Algae growth ($Growth_{Algae}$) is correlated in terms of nutrients concentration given by eq (38) [9] where (TotP) and (TotN) concentrations are given in milligrams per liter of phosphorus and nitrogen respectively.

$$\text{Growth}_{Alage}\left(\frac{g}{m^2 d}\right) = 0.418528\cdot\text{TotP} + 0.52762\cdot\text{TotN} + 0.225013\cdot\text{TotN}\cdot\text{TotP} - 0.20754\cdot\text{TotP}^2 - 0.03026\cdot\text{T} \quad (38)$$

The consumed rate of CO_2 (CO_{2comp}) due to algae growth is given by eq (39) [10].

$$CO_{2comp}\left(\frac{m^3}{d}\right) = 0.6565\cdot Growth_{Algae}\left(\frac{g}{m^2\, d}\right) + 5.0784 \quad (39)$$

In the transesterification reactor, the reaction yield is computed by eq (40) [11]. The range of operation of the variables for eq (40) are shown in Table S1.

(40)

Table S1.-Range of operation of the variables. Heterogeneous catalysis.

Variable	Lower bound	Upper bound
Temperature (ºC)	40	60
Ratio methanol (mol/mol)	6	12
Cat (%)	1	4

Vibrating Sieve

The model of the vibrating sieve is based on mass balance, assuming an isothermal operation.

$$m_{Sawdust}^{inlet} = m_{Sawdust}^{out\ to\ dryer} + m_{Sawdust}^{out\ to\ splitter} \tag{41}$$

$$m_{H_2O}^{inlet} = m_{H_2O}^{out\ to\ dryer} + m_{H_2O}^{out\ to\ splitter} \tag{42}$$

$$m_{Ash}^{inlet} = m_{Ash}^{out\ to\ dryer} + m_{Ash}^{out\ to\ splitter} \tag{43}$$

$$\sum_i m_i^{out\ to\ dryer} = \sum_i m_i^{inlet} \cdot \tau \tag{44}$$

$$m_{Ash}^{inlet} = m_{total}^{inlet} \cdot x_{Ash} \tag{45}$$

$$m_{H_2O}^{inlet} = m_{total}^{inlet} \cdot x_{H_2O} \tag{46}$$

$$m_{Sawdust}^{inlet} = m_{total}^{inlet} \cdot \left(1 - x_{H_2O} - x_{Ash}\right) \tag{47}$$

$$\sum_i m_i^{out\ to\ splitter} = \sum_i m_i^{inlet} \cdot (1-\tau) \quad (48)$$

Where:

- τ: Fraction of the incoming stream to the screen going to the dryer.
- x_{H_2O}: Water mass fraction in wet sawdust.
- x_{Ash}: Ash mass fraction in wet sawdust.

The vibrating sieve design process was carried out from the procedure described in literature [12]. The sieving area is determined as follows:

$$A_{sieving} = \frac{Q}{F_B \cdot C_R} \quad (49)$$

Where:

- $A_{sieving}$: Screening area (m2)
- Q: Mass rate of flow in sieving screen (t/h).
- F_B: Basic capacity (t/h/m2).
- C_R: Combined correction factor.

Power consumption was considered from commercial sieve data [13], see Table S2.

Table S2. Power consumed by different capacity of sieving screen.

Capacity of sieving screen (kg/h)	Power (kW)
250-350	150
350-600	300
600-700	420
700-1000	600
1000-1300	850
1300-2000	1500
2000-3000	2000
3000-3500	2200

Sawdust Dryer

The wet bulb temperature of flue gases was determined considering the same dependence of the wet bulb temperature of the air with temperature [14]. The maximum allowed combustion gas rate (ft/s) was determined by optimization setting as a constraint a maximum mass speed of the combustion gas of 5000 lb/ft²h. On the other hand, a maximum moisture content in sawdust of 10% was allowed.

$$m_{Sawdust}^{outlet\ to\ reactor} = m_{Sawdust}^{inlet\ from\ sieve} \tag{50}$$

$$m_{H_2O}^{outlet\ to\ reactor} = m_{H_2O}^{inlet\ from\ sieve} \cdot \left(1 - R_{H_2O}^{\%}\right) \tag{51}$$

$$m_{Ash}^{outlet\ to\ reactor} = m_{Ash}^{inlet\ from\ sieve} \tag{52}$$

$$m_{H_2O}^{outlet\ to\ sink} = \left(m_{H_2O}^{inlet\ from\ sieve} \cdot R_{H_2O}^{\%}\right) + m_{H_2O}^{inlet\ from\ furncae} \tag{53}$$

$$m_{CO_2}^{outlet\ to\ sink} = m_{CO_2}^{inlet\ from\ furncae} \tag{54}$$

$$m_{N_2}^{outlet\ to\ sink} = m_{N_2}^{inlet\ from\ furncae} \tag{55}$$

$$m_{O_2}^{outlet\ to\ sink} = m_{O_2}^{inlet\ from\ furncae} \tag{56}$$

Where:

- $R_{H_2O}^{\%}$: Percentage of water removal.

The energy balance in eq (57), was made considering that the dyer operates adiabatically, without heat losses.

$$\frac{qt}{m_s} = \left(C_{ps} \cdot (T_{sb} - T_{sa})\right) + X_a \cdot C_{pl} \cdot (T_v - T_{sa}) + \lambda \cdot (X_a - X_b) + \\ + X_b \cdot C_{pl} \cdot (T_{sb} - T_v) + (X_a - X_b) \cdot C_{pv} \cdot (T_{vb} - T_v) \tag{57}$$

Where:

- T_{sa}: Initial temperature of solids.
- T_{sb}: Final temperature of solids.
- T_v: Vaporization temperature of liquid to be removed.
- T_{vb}: Final temperature of steam.
- λ: Latent heat of vaporization.
- X_a: Initial moisture on day basis.
- X_b: Final moisture on dry basis.
- C_{ps},C_{pl},C_{pv}: Specific hear of solids, liquid to be removed and vapor respectively.
- m_s: Mass of dry solid.

For the dryer design, the number of heat transfer units (N_t) is determined by eq (58) [15], where the total flow of drying gas is computed by eq (59):

$$N_t = \frac{(T_{ha} - T_{hb})}{\frac{(T_{ha} - T_{wa}) - (T_{hb} - T_{wb})}{ln\left(\frac{T_{ha} - T_{wa}}{T_{hb} - T_{wb}}\right)}} \quad (58)$$

Where:

- T_{ha}: Initial drying agent temperature.
- T_{hb}: Final drying agent temperature.
- T_{wa}: Initial wet bulb temperature.
- T_{wb}: Final wet bulb temperature.

$$qt = m_g \cdot (1 + H_a) \cdot C_{sa} \cdot (T_{ha} - T_{hb}) \quad (59)$$

Where:

- m_g: Dry gas mass velocity.
- H_a: Initial moisture of dry gas.
- C_{sa}: Specific heat of inlet gas in dryer.

The dryer diameter (D_{Dryer}) is calculate as follows, where G is the drying agent mass rate:

$$D_{Dryer} = \sqrt{\frac{4 \cdot \left(\frac{m_g}{G}\right)}{\pi}} \quad (60)$$

The length of dryer (L_{Dryer}), and the power consumed per dryer [16] (P_D) are determined with eqs (61-63).

$$L_{Dryer} = \frac{qt}{0.125 \cdot \pi \cdot D_{Dryer} \cdot G^{0.67} \cdot \Delta T_{Dryer}} \quad (61)$$

$$P_D = 0.3 \cdot \frac{\pi \cdot {D_{Dryer}}^2 \cdot L_{Dryer}}{4} \quad (62)$$

$$\Delta T_{Dryer} = \frac{(T_{ha} - T_{wa}) - (T_{hb} - T_{wb})}{ln\left(\frac{T_{ha} - T_{wa}}{T_{hb} - T_{wb}}\right)} \quad (63)$$

Combustion Furnace

Atomic mass balance was carried out in order to model the combustion chamber, assuming a complete combustion of the sawdust, fame, and oil. A 65% of excess air was considered.

Carbon Balance

$$\left(7\cdot m_{Sawdust}^{inlet}\cdot\frac{Pm_C}{Pm_{Sawdust}}\right)+\left(19\cdot m_{FAME}^{inlet}\cdot\frac{Pm_C}{Pm_{FAME}}\right)+\left(57\cdot m_{Oil}^{inlet}\cdot\frac{Pm_C}{Pm_{Oil}}\right)=\left(1\cdot m_{CO_2}^{outlet}\cdot\frac{Pm_C}{Pm_{CO_2}}\right) \quad (64)$$

Oxygen Balance

$$\left(1\cdot m_{H_2O}^{inlet}\cdot\frac{Pm_O}{Pm_{H_2O}}\right)+\left(5\cdot m_{Sawdust}^{inlet}\cdot\frac{Pm_O}{Pm_{Sawdust}}\right)+\left(2\cdot m_{FAME}^{inlet}\cdot\frac{Pm_O}{Pm_{FAME}}\right)+\left(6\cdot m_{Oil}^{inlet}\cdot\frac{Pm_O}{Pm_{Oil}}\right)+\left(2\cdot m_{O_2}^{inlet}\cdot\frac{Pm_O}{Pm_{O_2}}\right)+\left(1\cdot m_{H_2O}^{inlet}\cdot\frac{Pm_O}{Pm_{H_2O}}\right)=\left(2\cdot m_{CO_2}^{outlet}\cdot\frac{Pm_O}{Pm_{CO_2}}\right)+\left(2\cdot m_{O_2}^{outlet}\cdot\frac{Pm_O}{Pm_{O_2}}\right)+\left(1\cdot m_{H_2O}^{outlet}\cdot\frac{Pm_O}{Pm_{H_2O}}\right) \quad (65)$$

Hydrogen Balance

$$\left(2\cdot m_{H_2O}^{inlet}\cdot\frac{Pm_H}{Pm_{H_2O}}\right)+\left(10\cdot m_{Sawdust}^{inlet}\cdot\frac{Pm_H}{Pm_{Sawdust}}\right)+\left(36\cdot m_{FAME}^{inlet}\cdot\frac{Pm_H}{Pm_{FAME}}\right)+\left(104\cdot m_{Oil}^{inlet}\cdot\frac{Pm_H}{Pm_{Oil}}\right)+\left(2\cdot m_{H_2O}^{inlet}\cdot\frac{Pm_H}{Pm_{H_2O}}\right)=\left(2\cdot m_{H_2O}^{outlet}\cdot\frac{Pm_H}{Pm_{H_2O}}\right) \quad (66)$$

$$m_{N_2}^{inlet}=m_{N_2}^{outlet} \quad (67)$$

$$m_{Ash}^{inlet}=m_{Ash}^{outlet} \quad (68)$$

Where:

- m_i^j: Mass flow of each component i in the stream j in the furnace.
- Pm_i: Molecular weight of each component i.

The energy balance was made considering an adiabatic operation conditions

$$\sum_j H_{inlet}^j=\sum_j H_{outlet}^j \quad (69)$$

Where:

- H^j: Total enthalpy of stream j involved in the furnace.

Liquefaction reactor

For the mass balances the following parameters were considered [17].

- Reaction temperature and pressure 150 °C and 1 atm.
- Liquefaction time of 45 minutes.
- Sawdust particle size: between 0.30 and 0.60 mm.
- Biomass / sulfuric acid ratio of 8.88: 1.
- Catalyst purity (98% sulfuric acid).
- Glycerol / biomass ratio of 5.04: 1.
- Liquefaction conversion: 99.7%.

The liquefaction reactor is based on the following mass and energy balances eq (70-77):

$$m_{Sawdust}^{outlet} = m_{Sawdust}^{inlet} \cdot (1 - R_{polyol}) \quad (70)$$

$$m_{Glycerol}^{outlet} = m_{Glycerol}^{inlet} \cdot (1 - R_{polyol}) \quad (71)$$

$$m_{Ash}^{outlet} = m_{Ash}^{inlet} \quad (72)$$

$$m_{H_2O}^{outlet} = m_{H_2O}^{inlet\ from\ sawdust} + m_{H_2O}^{inlet\ from\ sulfuric\ acid} \quad (73)$$

$$m_{H_2SO_4}^{outlet} = m_{H_2SO_4}^{inlet} \quad (74)$$

$$m_{Polyol}^{outlet} = R_{polyol} \cdot (m_{Sawdust}^{inlet} + m_{Glycerol}^{inlet}) \quad (75)$$

$$m_{CH_3OH}^{outlet} = m_{CH_3OH}^{inlet} \quad (76)$$

Where:

- m_i^j: Mass flow of each component i in the stream j in the liquefaction reactor.
- R_{polyol}: Ratio between polyol produced and sawdust and glycerol fed to the liquefaction reactor.

$$\Delta H_{heatreaction} \cdot m_{Total}^{outlet} + \sum_j H_{outlet}^j = \sum_j H_{inlet}^j \quad (77)$$

Where:

- H^j: Total enthalpy of stream j involved in the liquefaction reactor.
- $\Delta H_{heatreaction}$ = -186.72 kJ/kg. Liquefaction reaction heat [17].

The design of the liquefaction reactor was carried out employing the technical specifications of lab scale reactor used in the experimentations [17], see Table S3.

Table S3. Technical Specification of Laboratory Reactor needed to scale up.

**Technical specification of laboratory reactor for scale-up**	
High (m)	0.27
Diameter (m)	0.14

The scale-up ratio ($R_{reactor}$) was determined from the diameter of lab reactor and diameter required for the volume of the reactor in plant [18].

$$R_{reactor} = \frac{D_{f_{reactor}}}{D_{l_{reactor}}} \qquad (78)$$

Where:

- $D_{f_{reactor}}$: Diameter of reactor in plant.
- $D_{l_{reactor}}$: Diameter of reactor in laboratory.

The diameter of reactor in plat was determined using the volume of each reactor ($V_{reactor}$) and considering torispherical heads with a ratio of high/diameter of 1.93 [19].

$$V_{reactor} = \left(\frac{\pi \cdot D_{f_{reactor}}^2}{4} \cdot L_{f_{reactor}}\right) + \left(0.0809 \cdot D_{f_{reactor}}^3\right) + \left(0.0809 \cdot D_{f_{reactor}}^3\right) \qquad (79)$$

Where:

- $V_{reactor}$: Volume of reactor in plant.
- $L_{f_{reactor}}$: Height of reactor in plant.

Be known the ratio between height/diameter of reactor, the diameter of reactor will be determined by eq (79) to subsequently determine the height of reactor by the eq (80) [18].

$$L_{f_{reactor}} = R_{reactor} * L_{l_{reactor}} \qquad (80)$$

Where:

- $L_{l_{reactor}}$: Height of reactor in laboratory.

The power consumption ($P_{reactor}$) of the reactor agitators was calculated based on the following experimental values [17], using the eqs (81-83) [18,19].

- Mechanical stirrer with a stirring speed of 300-500 rpm throughout the reaction.
- Viscosity of the reaction mixture: 6 Pa·s.
- Mixing density: 0.93 g/cm3.
- Viscosity of the generated polyol (25°C): 6 Pa·s.
- Density of the generated polyol (25°C): 1.2-1.3 g/cm3.

$$P_{reactor} = n_{reactor} \cdot N_{p_{reactor}} \cdot \rho_{reactor} \cdot N_{reactor}^3 \cdot {D'_{reactor}}^5 \tag{81}$$

$$D'_{reactor} = R'_{reactor} \cdot L_{f_{reactor}} \tag{82}$$

$$N_{reactor} = N'_{reactor} \cdot \left(\frac{1}{R_{reactor}}\right)^{2/3} \tag{83}$$

Where:

- $n_{reactor}$: Number of impellers in reactor.
- $N_{p_{reactor}}$: Power number of reactor.
- $\rho_{reactor}$: Density of the generated polyol in reactor.
- $N_{reactor}$: Speed of mixing in reactor.
- $D'_{reactor}$: Diameter of impeller in the reactor.
- $R'_{reactor}$: Ratio between diameter of impeller and height of reactor [18].
- $N'_{reactor}$: Speed of mixing in lab reactor.

Centrifuge

The mass balances were carried out by eqs (84-85) assuming that all the solids (sawdust and ashes) that comes from the liquefaction reactor are removed, together with the 10% of glycerol, water, methanol and sulfuric acid as moisture [17]. In this way, the $f_{removed}$ for solids are 1 and for liquid compounds 0.1. Isothermal operation conditions were considered. The diameter of the centrifuge was determined by eq (86) [20]. The power consumed by the centrifuge was determined as a function of the volumetric flow [21].

$$\sum_i m_i^{outlet\ to\ sink} = \sum_i m_i^{inlet} \cdot f_{removed} \tag{84}$$

$$\sum_i m_i^{outlet\ to\ mixer} = \sum_i m_i^{inlet} \cdot (1 - f_{removed}) \qquad (85)$$

$$D_{centrifuge} = (0.3308 \cdot 3.6 \cdot Q_{centrifuge}) + (9.5092) \qquad (86)$$

Where:

- $D_{centrifuge}$: Diameter of centrifuge (inch).
- $Q_{centrifuge}$: Mass rate of flow in centrifuge (kg/s).
- m_i^i: Mass flow of each component i in the stream j in the centrifuge.

Extruder

The extruder was modelled based on the following mass balance, eq (87). In order to design the equipment was considered the data below [17]. The power consumptions for different capacities are represented in Table S4 [13].

- Rotation speed: 90 rpm
- Extruding temperature: 60 - 115 °C

$$\sum_i m_i^{outlet} = \sum_i m_i^{inlet} \qquad (87)$$

Where:

- m_i^i: Mass flow of each component i in the stream j in the extruder.

Table S4. Power to different capacity of extruders.

Capacity (kg/h)	Power (kW)
100 – 140	65 – 70
80 – 180	150
150 – 300	75 – 80
> 250	92

Mixer / Polymerization reactor

Assuming isothermal operating, only a mass balances is required for modelling, as following in eqs (88-89). A total conversion of the polyol and the starch to the polymer together with a mass ratio starch/polyol of 2.63 were considered [17].

$$\sum_i m_{i-\{polymer,\, polyol,\, starch\}}^{outlet} = \sum_i m_{i-\{polymer,\, polyol,\, starch\}}^{inlet\ from\ HX31} + \sum_i m_{i-\{polymer,\, polyol,\, starch\}}^{inlet\ from\ Tank1} \tag{88}$$

$$m_{polymer}^{outlet} = m_{polyol}^{inlet\ from\ HX31} + m_{starch}^{inlet\ from\ Tank1} \tag{89}$$

Where:

- m_i^i: Mass flow of each component i in the stream j in the mixer.

The design of the polymerization reactor was carried out employing the technical specifications of lab scale reactor used in the experimentations [17], see Table S5.

Table S5. Technical Specification of Lab Polymerization Reactor.

**Technical specification**	
High (m)	1.57
Diameter (m)	0.52

The scale-up ratio (R_{mixer}) was determined from the diameter of lab reactor and diameter required for the volume of the reactor in plant [18].

$$R_{mixer} = \frac{D_{f_{mixer}}}{D_{l_{mixer}}} \tag{90}$$

Where:

- $D_{f_{mixer}}$: Diameter of mixer in plant.
- $D_{l_{mixer}}$: Diameter of mixer in laboratory.

The diameter of reactor in plat was determined using the volume of each reactor (V_{mixer}) and considering torispherical heads with a ratio of high/diameter of 3 [19].

$$V_{mixer} = \left(\frac{\pi \cdot D_{f_{mixer}}^2}{4} \cdot L_{f_{mixer}}\right) + \left(0.0809 \cdot D_{f_{mixer}}^3\right) + \left(0.0809 \cdot D_{f_{mixer}}^3\right) \quad (91)$$

Where:

- V_{mixer}: Volume of mixer in plant.
- $L_{f_{mixer}}$: Height of mixer in plant.

Be known the ratio between height/diameter of reactor, the diameter of reactor will be determined by eq (91) to subsequently determine the height of reactor by the eq (92) [18].

$$L_{f_{mixer}} = R_{mixer} * L_{l_{mixer}} \quad (92)$$

Where:

- $L_{l_{mixer}}$: Height of mixer in laboratory.

The power consumption ($P_{reactor}$) of the reactor agitators was calculated based on the following experimental values [17], using the eqs (93-95) [18,19].

- Mechanical stirrer with a stirring speed of 300-500 rpm.
- Viscosity of mixture: 6 Pa.s.
- Mixing density: 0.93 g/cm3.

$$P_{mixer} = n_{mixer} \cdot N_{p_{mixer}} \cdot \rho_{mixer} \cdot N_{m_{mixer}}^3 \cdot D'^5_{m_{mixer}} \quad (93)$$

$$D'_{m_{mixer}} = R'_{mixer} \cdot L_{f_{mixer}} \quad (94)$$

$$N_{m_{mixer}} = N'_{m_{mixer}} \cdot \left(\frac{1}{R_{mixer}}\right)^{2/3} \quad (95)$$

Where:

- n_{mixer}: Number of impellers in mixer.
- $N_{p_{mixer}}$: Power number in mixer.
- ρ_{mixer}: Density of mixture.
- $N_{m_{mixer}}$: Speed of mixing in the mixer.
- $D'_{m_{mixer}}$: Diameter of impeller in mixer.

- R'_{mixer}: Ratio between diameter of impeller and height of reactor [18].
- N'_{mixer}: Speed of mixing in lab reactor.

Distillation columns

The model for the three distillation columns is represented by eqs (96-102) [22].

$$\sum_i y_{i,s} \cdot P_{v,i} \cdot (T_s) = P_{Column} \qquad s \in \{feed, distillate, bottoms\} \tag{96}$$

$$fc_{i,feed} = fc_{i,distillate} + fc_{i,bottoms} \tag{97}$$

$$fc_{i,distillate} = \eta_i \cdot fc_{i,feed} \tag{98}$$

$$Q_{Cond,total} = (1 + \mathrm{R}) \cdot \sum_i \lambda_{i,\, distillate} \cdot (T_{distillate}) \cdot fc_{i,distillate} \tag{99}$$

$$Q_{Cond,partial} = (\mathrm{R}) \cdot \sum_i \lambda_{i,\, distillate} \cdot (T_{distillate}) \cdot fc_{i,distillate} \tag{100}$$

$$Q_{boiler,partial} = (1 + \mathrm{R}) \cdot \sum_i fc_{i,distillate} \sum_i \lambda_{i,\, bottoms} \cdot (T_{bottoms}) \cdot x_{i,bottoms} \tag{101}$$

$$ln(P_v[mmHg]) = \mathrm{A} - \frac{B}{C + T[°\mathrm{C}]} \tag{102}$$

Costing and Project Evaluation

The economic assessment was made following the factorial method proposed by Towler and Sinnott [23]. The fixed capital is computed from the price of the main equipment of the facility (PCE). The correlations for the equipment can be found in the supplementary materials of Almena and Martín [24] , Roldán-San Antonio [13] and in the "Appendix A. Additional equipment cost correlations" of this Supplementary material [9,25].

The total physical plant costs (PPC) are determined from the sum of the following items in the Table S5, assuming a facility which works with solids and fluids.

Table S5.Total physical plant costs.

Items	Fraction of PCE
Equipment cost (PCE)	1.00
Equipment erection	0.45
Piping	0.45
Instrumentation	0.15
Electrical	0.10
Buildings, process	0.10
Storages	0.2
Site development	0.05
Ancillary buildings	0.20

The indirect costs plant costs are computed as the sum of the following items in the Table S6. In this way, the fixed capital is the sum of the total physical plant costs and the indirect costs.

Table S6. Indirect costs.

Items	Fraction of PPC
Design and Engineering	0.25
Contractor's fee	0.05
Contingency	0.10

The working capital is assumed as 5% of the fixed capital due to a large part of the raw material are produced in the facility. In this way, the sum of the fixed and working capital gives as the total investment capital of the facility.

In relation to the variable costs of the facility, they have been calculated as the sum of the costs from the raw materials, the utilities, and miscellaneous materials (10% of Maintenance item). On the other hand, the fixed costs of the facility are computed as the sum of the following items in Table S7.

Table S7. Fixed Costs.

Items	Fraction

Maintenance	0.1 of Fixed Capital
Operating Labour	37000$/yr. considering 6 person per shift. A total of 4 shifts [26,27].
Laboratory	0.23 of Operating Labour
Supervision	0.20 of Operating Labour
Plant Overheads	0.50 of Operating Labour
Capital Charges	0.10 of Fixed Capital
Insurance	0.010 of Fixed Capital
Local Taxes	0.020 of Fixed Capital
Equipment amortization	FA of 10.59

The sum of variable and fixed costs conforms direct operation costs. Other expenses as general overheads and research and development are considered as the 30% of the direct operation costs. In this way, the total annual production costs are the sum of this last item and direct operation costs.

The unity cost of the biopolymer is computed as the ratio of the annual production costs and the polymer annual production.

Appendix A. Additional equipment cost correlations

$$C_{digester}(\$) = 1475100 \cdot [flow\ of\ waste\ (kg/s)]^{0.82227} \qquad (103)$$

$$C_{evaporator}(\$) = 2447.5 \cdot Area\ (ft^2) + 29934 \qquad (104)$$

Heat Exchanger Matches

Table S8 and Table S9 show the specification of the stream matches for energy integration for the manure and sludge case studies respectively. Moreover, Figure S1 and Figure S2 show graphically the defined stream matches for the manure and sludge respectively.

Table S8. Stream matches for Manure case study.

					HOT		COLD	
**Hot stream**	**Number Hot Stream**	**Cold Matches**	**Number Cold Matches**	**Heat Exchanged (kJ/s)**	**Inlet temperature (Celsius)**	**Outlet temperature (Celsius)**	**Inlet temperature (Celsius)**	**Outlet temperature (Celsius)**
HX4	1	HX24	7	5,198	990	285	274	274
		HX22	8	1,581	285	70	60	227
HX1	2	HX3	6	119	275	243	233	251
HX26	3	HX3	6	5	275	261	252	252

HX23	4	HX3	6	808	244	244	109	233
		TransR	11	4,276	244	244	60	60
		HX9	9	854	244	244	105	200
		HX20	10	680	244	244	150	150
HX25	5	HX22	8	55	243	237	227	233

Table S9. Stream matches for Sludge case study.

					**HOT**		**COLD**	
**Hot stream**	**Number Hot Stream**	**Cold Matches**	**Number Cold Matches**	**Heat Exchanged (kJ/s)**	**Inlet temperature (Celsius)**	**Outlet temperature (Celsius)**	**Inlet temperature (Celsius)**	**Outlet temperature (Celsius)**
HX4	1	HX24	5	4,879	920	284	274	274
		HX3	4	1,126	284	136	106	274
HX1	2	HX22	6	99	275	250	234	244
HX23	3	TransR	9	4,276	244	244	60	60
		HX22	6	1,649	244	244	60	234
		HX9	7	896	244	244	105	200
		HX20	8	680	244	244	150	150

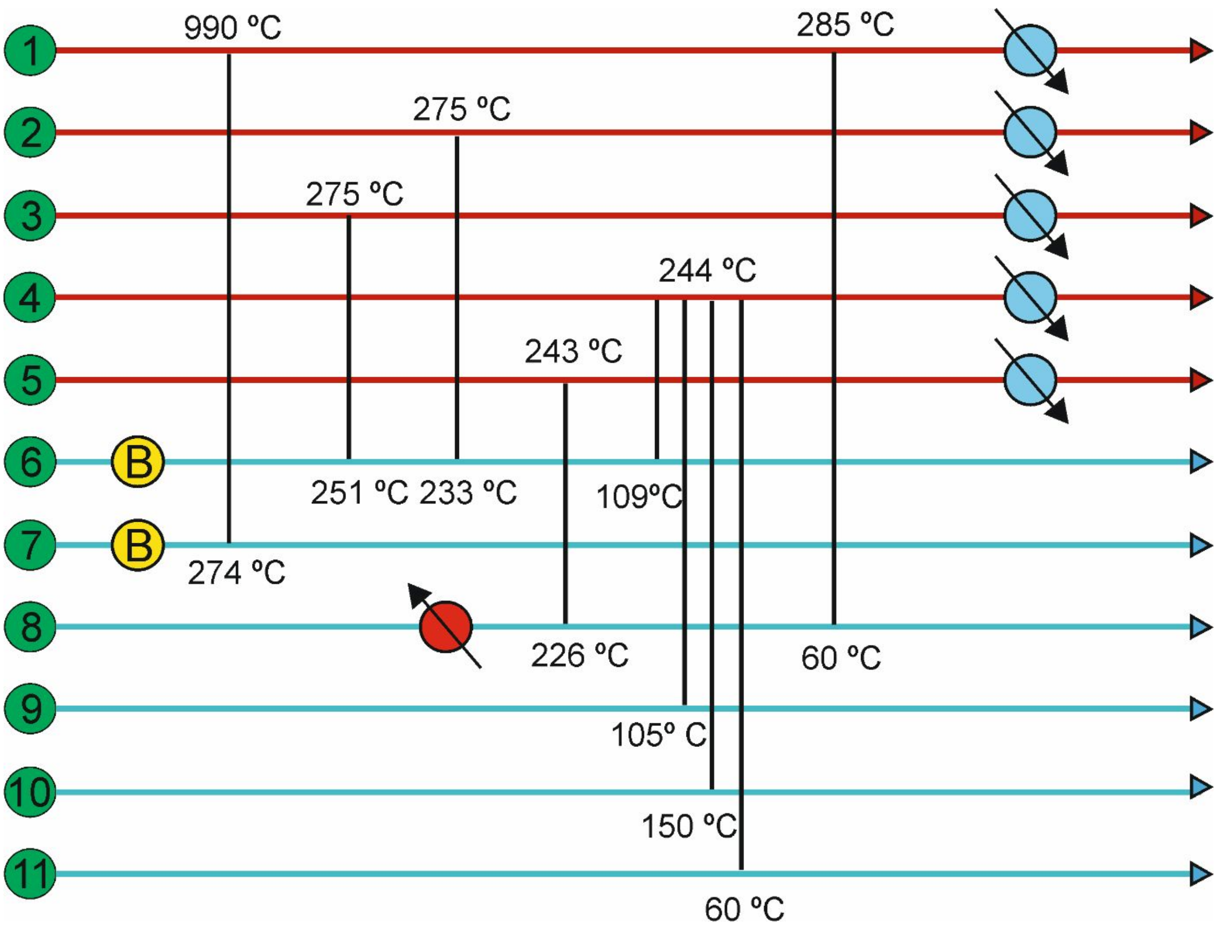

Figure S1. Stream matches for Manure case study.

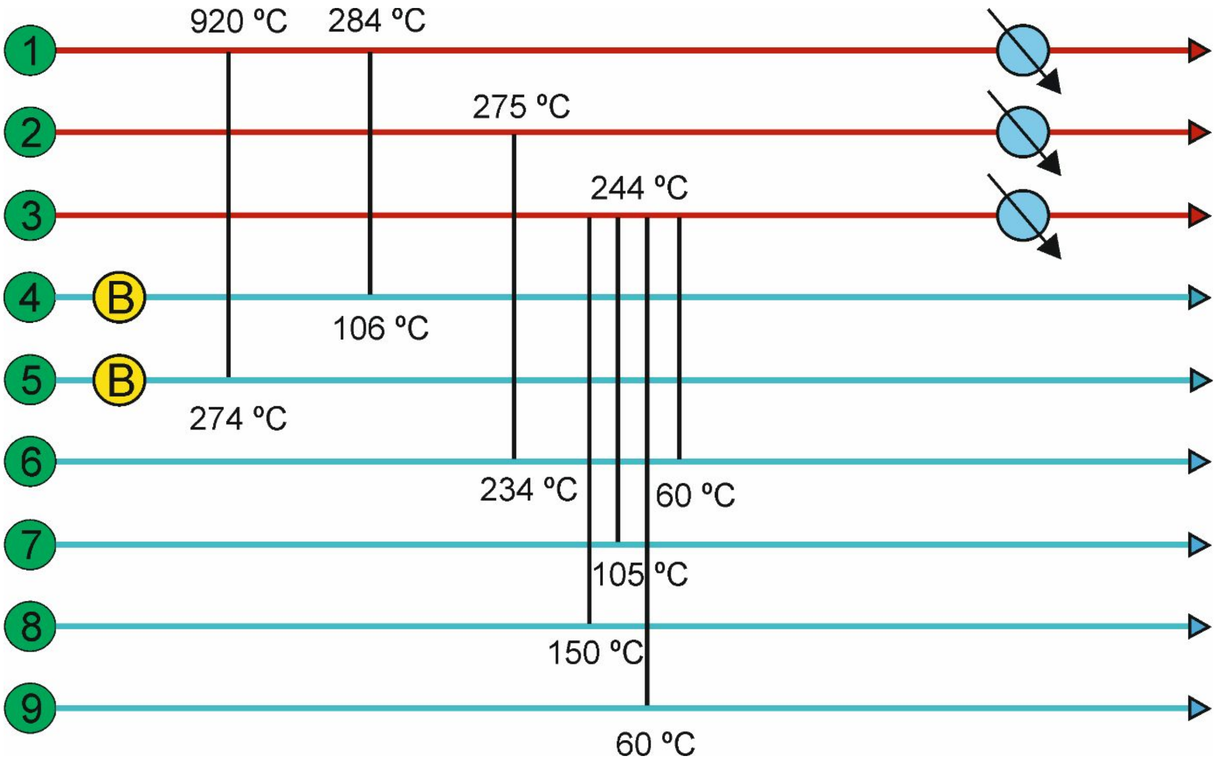

Figure S2. Stream matches for Sludge case study.

Accuracy of the Results

The results presented in the work are provided from the facility model resolution as well as the design of each of the equipment involved and the study of the economic evaluation of the integrated plant. The integrated plant model is constituted by the sub-models of each equipment, based on the physical principles of mass and energy conservation as well as subrogated models, thermodynamic relationships, and experimental data to evaluate their performances and yields. In relation to the modelling of the anaerobic digester, average biogas composition bounded values from validated literature [2] were employed to evaluate the performance of the digester, being a reference for other works based on biogas production [3,5]. For a more precise calculation, the reaction enthalpy associated with the metabolism of the anaerobic microorganisms and the composition of the resulting biogas for a specific composition of the feed would be required. However, these are highly specific data non available, being the reason to use average values of yields. Even so, the results obtained are consistent with the literature. The reformer and the methanol synthesis reactor are equilibrium reactors based on empirical correlations for the determination of the equilibrium constants of the reactions involved. In this way, the results obtained from the syngas production are validated by the use of thermodynamic correlations from previous studies for the

reactions (21-23) [7,28]. In the same way for the synthesis methanol reactions (32) [29,30]. On the other hand, the performance of the transesterification reactor is calculated on the basis of an empirical model obtained by the response surface methodology, represented by eq. (40) [11]. This model has a significance level probability less than 0.0001, together with a coefficient of determination equal to 0.99, showing a high statistical significance and good fit. Since the levels used in eq. (40) are within the ranges of the empirical model that was developed, the results from this model are considered highly significant. In addition, the yield model of the sawdust liquefaction reaction provided by the eq. (12) in the main text, as well as the mass ratio constraints for the polyol and polymer synthesis for the reaction conditions specified in the work were obtained from laboratory scale experimental data together with the technical design specifications of the equipment used in the experimentations [17]. In this way, the scale design of the equipment associated with the biodegradable plastic synthesis section were carried out. For reformer, methanol synthesis and transesterification sections, the designs of the involved equipment were performed following the methodology of previous authors [19,31–34]. With the level of detail considered at the design stage of the integrated facility, an estimated maximum error of 30% is assumed in the economic evaluation study [32].

References

(1) Gunaseelan, V. N. Anaerobic Digestion of Biomass for Methane Production: A Review. *Biomass and bioenergy* **1997**, *13* (1–2), 83–114. https://doi.org/10.1016/S0961-9534(97)00020-2.

(2) Al Seadi, T.; Rutz, D.; Prassl, H.; Köttner, M.; Finsterwalder, T.; Volk, S.; Janssen, R. *Biogas Handbook*; University of Southern Denmark Esbjerg, 2008.

(3) León, E.; Martín, M. Optimal Production of Power in a Combined Cycle from Manure Based Biogas. *Energy Convers. Manag.* **2016**, *114*, 89–99. https://doi.org/10.1016/J.ENCONMAN.2016.02.002.

(4) Elsholkami, M.; Elkamel, A.; Vargas, F. Optimized Integration of Renewable Energy Technologies into Alberta's Oil Sands Industry. *Comput. Chem. Eng.* **2016**, *90*, 1–22. https://doi.org/10.1016/J.COMPCHEMENG.2016.03.028.

(5) Hernandez, B.; Leon, E.; Martin, M. Bio-Waste Selection and Blending for the Optimal Production of Power and Fuels via Anaerobic Digestion. *Chem. Eng. Res. Des.* **2017**, *121*, 163–172. https://doi.org/10.1016/j.cherd.2017.03.009.

(6) Defra. *Fertiliser Manual (RB209) - Cattle slurry and dirty water - total and available nutrients*.

http://adlib.everysite.co.uk/adlib/defra/content.aspx?id=2RRVTHNXTS.88UF9N65FWLCJ (accessed 2022-12-13).

(7) Luyben, W. L. Design and Control of the Dry Methane Reforming Process. *Ind. Eng. Chem. Res.* **2014**, *53* (37), 14423–14439. https://doi.org/10.1021/ie5023942.

(8) Roh, H.-S.; Lee, D. K.; Koo, K. Y.; Jung, U. H.; Yoon, W. L. Natural Gas Steam Reforming for Hydrogen Production over Metal Monolith Catalyst with Efficient Heat-Transfer. *Int. J. Hydrogen Energy* **2010**, *35* (4), 1613–1619. https://doi.org/10.1016/j.ijhydene.2009.12.051.

(9) Hernández, B.; Martín, M. Optimal Integrated Plant for Production of Biodiesel from Waste. *ACS Sustain. Chem. Eng.* **2017**, *5* (8), 6756–6767. https://doi.org/10.1021/acssuschemeng.7b01007.

(10) Sazdanoff, N. Modeling and Simulation of the Algae to Biodiesel Fuel Cycle, The Ohio State University, 2006.

(11) Martín, M.; Grossmann, I. E. Simultaneous Optimization and Heat Integration for Biodiesel Production from Cooking Oil and Algae. *Ind. Eng. Chem. Res.* **2012**, *51* (23), 7998–8014. https://doi.org/10.1021/ie2024596.

(12) Gupta, A.; Yan, D. S. Introduction to Mineral Processing Design and Operation. *Perth, Aust.* **2006**, 564–570.

(13) Roldán-San Antonio, J. E.; Martín-Hernández, E.; Briones, R.; Martín, M. Process Design and Scale-up Study for the Production of Polyol-Based Biopolymers from Sawdust. *Sustain. Prod. Consum.* **2021**, *27*, 462–470. https://doi.org/10.1016/J.SPC.2021.01.015.

(14) Estrada-Jaramillo, M.; Vera-Romero, I.; Martínez-Reyes, J.; Ortíz-Soriano, A.; Barajas-Ledesma, E. Empirical Model to Calculate the Thermodynamic Wet-Bulb Temperature of Moist Air. *Engineering* **2014**, *2014*. https://doi.org/10.4236/eng.2014.69052.

(15) McCabe, W. L.; Smith, J. C.; Harriot, P. *Operaciones Unitarias En Ingeniería Química.*; McGraw Hill, 1994.

(16) Van't Land, C. M. *Drying in the Process Industry*; John Wiley & Sons, 2011.

(17) CIPA. *Biodegradable Polymer Based Materials Using Wood Residues of SME from Wood Industry in Chile's Biobío Region R15F10009*; 2017.

(18) Coker, A. K. *Modeling of Chemical Kinetics and Reactor Design*; Gulf Professional Publishing, 2001.

(19) Couper, J. R.; Penney, W. R.; Fair, J. R.; Walas, S. M. *Chemical Process Equipment: Selection and Design*; Butterworths Series in Chemical Engineering: Boston, 1990.

(20) Martín-Hernández, E.; Sampat, A. M.; Zavala, V. M.; Martín, M. Optimal Integrated Facility for Waste Processing. *Chem. Eng. Res. Des.* **2018**, *131*, 160–182. https://doi.org/10.1016/j.cherd.2017.11.042.

(21) Szepessy, S. Low Energy Consumption of High-Speed Centrifuges. *Chem. Eng. Technol.* **2018**, *vol.41* (12), 2375–2384. https://doi.org/https://doi.org/10.1002/ceat.201800292.

(22) Martín, M.; Redondo, J.; Grossmann, I. E. Optimal Integrated Facility for Oxymethylene Ethers Production from Methanol. *ACS Sustain. Chem. Eng.* **2020**, *8* (16), 6496–6504. https://doi.org/10.1021/acssuschemeng.0c01127.

(23) Towler, G.; Sinnott, R. *Chemical Engineering Design: Principles, Practice and Economics of Plant and Process Design*; Butterworth-Heinemann, 2012.

(24) Almena, A.; Martín, M. Technoeconomic Analysis of the Production of Epichlorohydrin from Glycerol. *Ind. Eng. Chem. Res.* **2016**, *55* (12), 3226–3238. https://doi.org/10.1021/acs.iecr.5b02555.

(25) Matches. *Matches' 275 Equipment Cost Estimates.* https://www.matche.com (accessed 2022-03-24).

(26) Anderson, J. Determining Manufacturing Costs. *CEP* **2009**, 27–31.

(27) Zippia. *Chemical Operator Salary*. https://www.zippia.com/chemical-operator-jobs/salary/ (accessed 2022-12-15).

(28) De Groote, A. M.; Froment, G. F. Reactor Modeling and Simulations in Synthesis Gas Production. *Rev. Chem. Eng.* **1995**, *11* (2), 145–183. https://doi.org/10.1515/REVCE.1995.11.2.145.

(29) Bissett, L. Equilibrium Constants for Shift Reactions. *Chem. Eng.* **1977**, *84(21)*, 155–156.

(30) Cherednichenko, V. . Dissertation, Karpova, Physico Chemical Institute, Moscow, U.S.S.R.; 1953.

(31) Baasal, W. D. *Preliminary Chemical Engineering Plant Design*; Springer Science & Business Media: New York, 1989.

(32) Sinnottt, R. K. *Chemical Engineering Design: Volume 6*, 3rd ed.; Oxford: Elsevier Butterworth-Heinemann, 1999.

(33) Branan, C. *Rules of Thumb for Chemical Engineers: A Manual of Quick*, 2nd ed.; Gulf Publishing Company: Texas, 1998.

(34) Douglas, J. M. *Conceptual Design of Chemical Processes*; McGraw-Hill: New York, 1988; Vol. 1110.
